# Incorporation of Sulfonamide Moiety into Biguanide Scaffold Results in Apoptosis Induction and Cell Cycle Arrest in MCF-7 Breast Cancer Cells

**DOI:** 10.3390/ijms22115642

**Published:** 2021-05-26

**Authors:** Magdalena Markowicz-Piasecka, Karol Sadowski, Johanna Huttunen, Joanna Sikora, Kristiina M. Huttunen

**Affiliations:** 1 Laboratory of Bioanalysis, Department of Pharmaceutical Chemistry, Drug Analysis and Radiopharmacy, Medical University of Lodz, ul. Muszyńskiego1, 90-151 Lodz, Poland; joanna.sikora@umed.lodz.pl; 2 Students Research Group, Department of Pharmaceutical Chemistry, Drug Analysis and Radiopharmacy, Medical University of Lodz, ul. Muszyńskiego1, 90-151 Lodz, Poland; karol.sadowski1@stud.umed.lodz.pl; 3 School of Pharmacy, Faculty of Health Sciences, University of Eastern Finland, Yliopistonranta 1C, POB 1627, 70211 Kuopio, Finland; johanna.huttunen@uef.fi (J.H.); kristiina.huttunen@uef.fi (K.M.H.)

**Keywords:** metformin, sulfonamides, apoptosis, cellular uptake, cell cycle arrest

## Abstract

Metformin, apart from its glucose-lowering properties, has also been found to demonstrate anti-cancer properties. Anti-cancer efficacy of metformin depends on its uptake in cancer cells, which is mediated by plasma membrane monoamine transporters (PMAT) and organic cation transporters (OCTs). This study presents an analysis of transporter mediated cellular uptake of ten sulfonamide-based derivatives of metformin in two breast cancer cell lines (MCF-7 and MDA-MB-231). Effects of these compounds on cancer cell growth inhibition were also determined. All examined sulfonamide-based analogues of metformin were characterized by greater cellular uptake in both MCF-7 and MDA-MB-231 cells, and stronger cytotoxic properties than those of metformin. Effective intracellular transport of the examined compounds in MCF-7 cells was accompanied by high cytotoxic activity. For instance, compound **2** with *meta*-methyl group in the benzene ring inhibited MCF-7 growth at micromolar range (IC_50_ = 87.7 ± 1.18 µmol/L). Further studies showed that cytotoxicity of sulfonamide-based derivatives of metformin partially results from their ability to induce apoptosis in MCF-7 and MDA-MB-231 cells and arrest cell cycle in the G0/G1 phase. In addition, these compounds were found to inhibit cellular migration in wound healing assay. Importantly, the tested biguanides are more effective in MCF-7 cells at relatively lower concentrations than in MDA-MB-231 cells, which proves that the effectiveness of transporter-mediated accumulation in MCF-7 cells is related to biological effects, including MCF-7 cell growth inhibition, apoptosis induction and cell cycle arrest. In summary, this study supports the hypothesis that effective transporter-mediated cellular uptake of a chemical molecule determines its cytotoxic properties. These results warrant a further investigation of biguanides as putative anti-cancer agents.

## 1. Introduction

Drug repositioning or repurposing, in contrast to a drug discovery process, is an effective approach to explore a new biological activity and identify novel indications for already well-known drugs [1]. There are numerous examples of effective drug repurposing with two well-known cases—anti-Parkinson amantadine, initially developed for influenza, and anti-HIV zidovudine, originally developed as an anti-cancer drug [2]. Metformin, a first-line oral agent for the treatment of type 2 diabetes (T2DM), is also a good example of drug which is tested in a wide variety of ailments, including pre-diabetes, gestational diabetes, polycystic ovary syndrome as well as in the treatment or prevention of pre-eclampsia [3]. Apart from the above-mentioned applications related to its glucose-lowering properties, the drug is characterized by multidirectional biological activity which contributes to favorable effects on the mortality rate in diabetic patients and improves the serum lipids profile, blood coagulation, and function of endothelium [4].

Beneficial characteristics of metformin caused an increased scientific interest due to its potential application of metformin as an anti-cancer agent. The majority of preclinical studies have consistently shown promising anti-proliferative effects of the drug in some cancer cell lines, and this has also been replicated to some degree in in vivo studies [5,6,7]. Metformin was found to suppress growth of several cancer cell lines, including breast, melanoma, lung cancer, endometrial cancer, leukemia or colorectal carcinoma [8]. Apart from the inhibitory effect on cancer cell growth, metformin was also found to reduce the risk of some types of cancers, and improve cancer prognosis [9]. Importantly, clinical data suggest that metformin may decrease cancer-related mortality, improve response to radiotherapy [10] and chemotherapy [11], and also reduce the malignancy of cancer [12]. Breast cancer is the malignant neoplasia with the highest prevalence. With regards to mortality, it occupies the second position. Due to these facts, the scientists concentrated their efforts on the evaluation of metformin therapeutic potential in this type of cancer [13,14,15]. For instance, Jiralerspong et al. [16] reported that diabetic patients receiving neoadjuvant chemotherapy demonstrated a greater response rate (24% in the metformin group vs. 8% in the non-metformin group) compared with patients who were administered other anti-diabetic drugs. Furthermore, it was shown that metformin synergistically acts with chemotherapeutic agents and specifically targets breast cancer stem cells [17].

Effects of metformin were also comprehensively studied using in vitro cellular models. The biological activity of metformin in MCF-7 and MDA-MB-231 cells has been particularly well examined. These two cell lines are frequently chosen because they correspond to different subtypes of breast cancer: luminal (positive for estrogen and progesterone receptors and negative for HER-2 receptor) and triple negative (TNBC), respectively [18]. Marinello et al. (2020) reported that clinical concentrations of metformin decrease the metabolic activity of MCF-7 cells; however, cell death and decreased cellular proliferation were observed at higher experimental concentrations (1–5 mM). In the case of MDA-MB-231 cells, death and decreased proliferation were reported only at higher doses. It was also found that metformin reduces cell survival by increasing the production of reactive oxygen species (ROS), which induce DNA damage, apoptosis [15] and necroptosis [13]. Further studies of Marinello’s team [18] confirmed that metformin upregulates genes involved in oxidative stress generation and apoptosis, and downregulates genes associated with metastasis in MCF-7 cells. In MDA-MB-231 cells, metformin downregulated genes involved in cell invasion, viability and proliferation. However, metformin does not cause the same anti-proliferative effects on all types of breast cancer cells since the effectiveness of growth inhibitory properties of the drug depends on the cellular uptake, which in turn depends on the expression of organic cation transporters (OCTs) on the cell surface [19].

Multispecific organic cation transporters are encoded by the *SLC22* gene family, and consist of three closely related subtypes: OCT1 (*SLC22A1*), OCT2 (*SLC22A2*), and OCT3 (*SLC22A3*). OCTs are expressed in several tissues, and mediate the intracellular transport of various endogenous and exogenous positively charged compounds. For instance, OCT1, located in the sinusoidal membrane of hepatocytes, has been found to act as the main carrier responsible for the uptake of metformin in the liver. In addition, the elimination of metformin is also transporter-dependent since the uptake of metformin from circulation into renal epithelial cells is primarily facilitated by OCT2, which is expressed at the basolateral membrane in renal tubules [20]. Apart from OCTs, other transporters such as plasma membrane monoamine transporter (PMAT; *SLC29A4*) and multidrug and toxin extrusion 1 and 2 (MATE 1, 2; *SLC47A1*, *SLC47A2*) participate in the cellular uptake of metformin, including absorption in the intestine. The above-mentioned transporters are not only involved in the pharmacokinetic properties of metformin but were also reported to contribute to variability in drug response, and clinically relevant drug-drug interactions [21,22].

Metformin is characterized by unfavorable physico-chemical properties with acid dissociation constant values (pKa) of 2.8 and 11.5. This contributes to the occurrence of metformin as hydrophilic cationic species at physiological pH values. The higher pKa value makes metformin a stronger base than most other basic drugs with less than 0.01% unionized in blood [23]. Therefore, there is a strong need to modify the structure of the biguanide skeleton of metformin to improve the pharmacokinetics of the drug. Modification of the metformin scaffold was also found to be promising in the improvement of anti-cancer properties. For instance Cheng et al. reported a synthesis of several analogues of metformin with varying alkyl chain lengths, containing a triphenylphosphonium cation (TPP+) which were found to be far more efficient than metformin itself regarding inhibition of the growth of pancreatic ductal adenocarcinoma (PDAC) [24]. The compound possessing 10-carbon aliphatic chains (Mito-Met10) was found particularly effective since it triggered G1 cell cycle phase arrest in PDAC cells, enhanced their radiosensitivity and more potently abrogated PDAC growth in preclinical mouse models, compared to metformin. Moreover, our previous studies revealed that chemical modification of the metformin scaffold into sulfenamides [25] or halogenated sulfonamides [26] improves OCT-mediated cellular uptake in breast cancer cells, and subsequently leads to greater inhibition of their growth.

Herein, we present chemical modification of the metformin backbone into more lipophilic sulfonamides with methyl substituents in the aromatic ring as a promising approach to target OCTs, improve cellular uptake and potentiate anti-cancer effects. The uptake of ten sulfonamide metformin derivatives differing with an alkyl substituent in the benzene ring (compounds **1**–**10**, Table 1) was comprehensively determined in two human breast cancer cell lines, MCF-7 and MDA-MB-231. Furthermore, the effects of metformin derivatives on the inhibition of cancer cell growth were reported. A few compounds were selected for determination of their mechanism of action based on the results of intracellular uptake and cytotoxicity screening. Hence, this study illustrates how the chemical transformation of the biguanide scaffold into sulfonamides, subsequent greater affinity towards OCTs, and OCT-mediated cellular uptake can improve cytotoxicity of the parent drug, metformin.

## 2. Results and Discussion

### 2.1. Cellular Uptake of Metformin Derivatives

#### 2.1.1. General Characterization

The first step of the studies included establishing a relationship between cytotoxic effects of the examined compounds and their cellular uptake in MCF-7 and MDA-MB-231 cells. Both examined cell lines were previously characterized according to the expression of OCT, PMAT and MATE transporters [26]. Among OCT transporters, OCT3 were predominant in MDA-MB-231 cells, while in the case of MCF-7, we were not able to detect any of OCT transporters. In the case of MATE transporters similar significant expression of MATE 1 in both cell lines was reported [26]. These results are in agreement with outcomes of Cai et al. [19] who also concluded that there is a relationship between the presence of cation-selective transporters, the effectiveness of metformin uptake and its antiproliferative activity.

**Figure 1:**
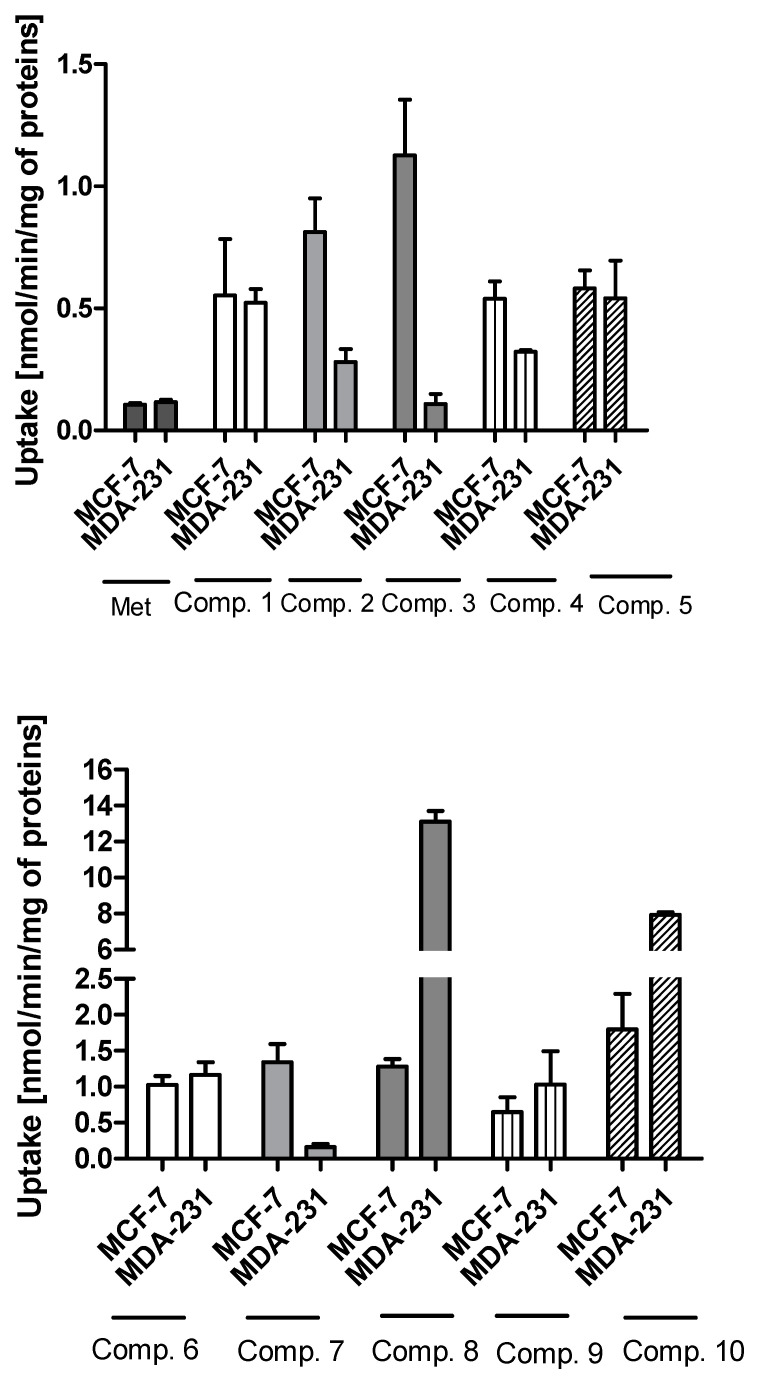
The uptake of sulfonamide derivatives of metformin (**1**–**10**) into MCF-7 and MDA-MB-231 cells at 800 μmol/L concentration after 10 min incubation at 37 °C. Results of metformin uptake in MCF-7 and MDA-MB-231 cells were presented previously [25].

The uptake studies enable one to assess how effectively metformin derivatives are transported into MCF-7 and MDA-MB-231 cells. These studies were performed at concentrations ranging from 10 to 2000 µmol/L. We chose one representative concentration of 800 µmol/L (Figure 1) to present the difference in the uptake of metformin derivatives in both cell lines. As seen in Figure 1, compound **1** without any substituent in aromatic ring showed similar uptake in MCF-7 and MDA-MB-231 cells (0.553 ± 0.231 nmol/min/mg of proteins in MCF-7, and 0.523 ± 0.056 nmol/min/mg of proteins). Mono-methyl benzenesulfonamides (compounds **2**–**4**) were uptaken more efficiently in MCF-7 cells than in MDA-MB-231 cells. For instance, the uptake of compound **2** was 0.813 ± 0.138 nmol/min/mg of proteins, and this value was approximately three-fold higher than that in MDA-MB-231 cells (0.280 ± 0.054 nmol/min/mg of proteins). However, these results were greater than those obtained for metformin (0.107 ± 0.006 and 0.117 ± 0.010 nmol/min/mg of proteins in MCF-7 and MDA-MB-231 cells, respectively) [25]. Even more considerable differences in the cellular uptake were reported in the case of compound **3** (1.127 ± 0.121 nmol/min/mg of proteins in MCF-7 vs. 0.109 ± 0.041 nmol/min/mg of proteins in MDA-MB-231 cells). The greater uptake of these compounds probably stems from their higher affinity towards PMAT transporters, whose expression is approximately three-fold higher in MCF-7 cells than in MDA-MB-231 cells [26]. In turn, compound **5** with *p*-propyl substituent was transported into MCF-7 and MDA-231 cells at a comparable rate (0.582 ± 0.074 nmol/min/mg proteins, and 0.542 ± 0.154 nmol/min/mg protein).

In the case of sulfonamides with two methyl groups, we observed significant differences in their cellular uptake in both cell lines. For instance, compound **6** with 2,4-dimethyl groups in the aromatic ring presented a similar ‘pattern’ of uptake in MCF-7 and MDA-MB-231 cells. On the other hand, compound **7** (2,5-dimethyl groups) was transported into MCF-7 cells approximately eight-fold more efficiently than in MDA-MB-231 cells. A different situation has been observed for compounds **8**, **9** and **10**, which were characterized by greater uptake in MDA-MB-231 cells than MCF-7 cells. For instance, the uptake rate of compound **8** was 13.102 ± 0.607 nmol/min/mg protein in MDA-MB-231 cells, while in MCF-7, it was 1.280 ± 0.104 nmol/min/mg protein. Compound **10** with 2,4,6-trimethyl substituents in the aromatic ring was characterized by 4.4-fold greater uptake in MDA-MB-321 cells than in MCF-7 cells. Based on these results, we presume that some of these compounds might utilize OCT3 transporters since in our previous studies [26] OCT3 were found to be predominant transporters in MDA-MB-231 cells.

#### 2.1.2. Kinetic Analysis of Sulfonamide Uptake

The obtained relationship between the concentration of the test compound and its uptake in cells were used to plot the Michaelis-Menten curves and to calculate the basic kinetic parameters of the ongoing processes. These results of kinetic parameters (K_m_, V_max_) together with the efficacy of cellular uptake (V_max_/K_m_) are presented in Table 2.

Concentrations at which the capacity of transporters and subsequent cellular uptake in MCF-7 cells reach the maximum value are various, depending on the compounds, and range from a few hundred micromoles (comp. **3**) to several thousand µM (comp. **1**, **2**). In the case of compound **4**, the K_m_ and V_max_ parameters could not be calculated, since the uptake was linear over the studied concentration range and no transporter saturation was observed. Based on the presented results we can conclude that all studied compounds exhibit greater efficacy of intracellular transport in MCF-7 cells in comparison to metformin. A similar conclusion can be made for the uptake of studied compounds in MDA-MB-231 cells, since most of the sulfonamides were found to be transported more effectively than metformin. For instance, compound **10** with three methyl groups in the aromatic ring was uptaken in MDA-MB-231 cells approximately 70-fold greater than metformin.

**Figure 2:**
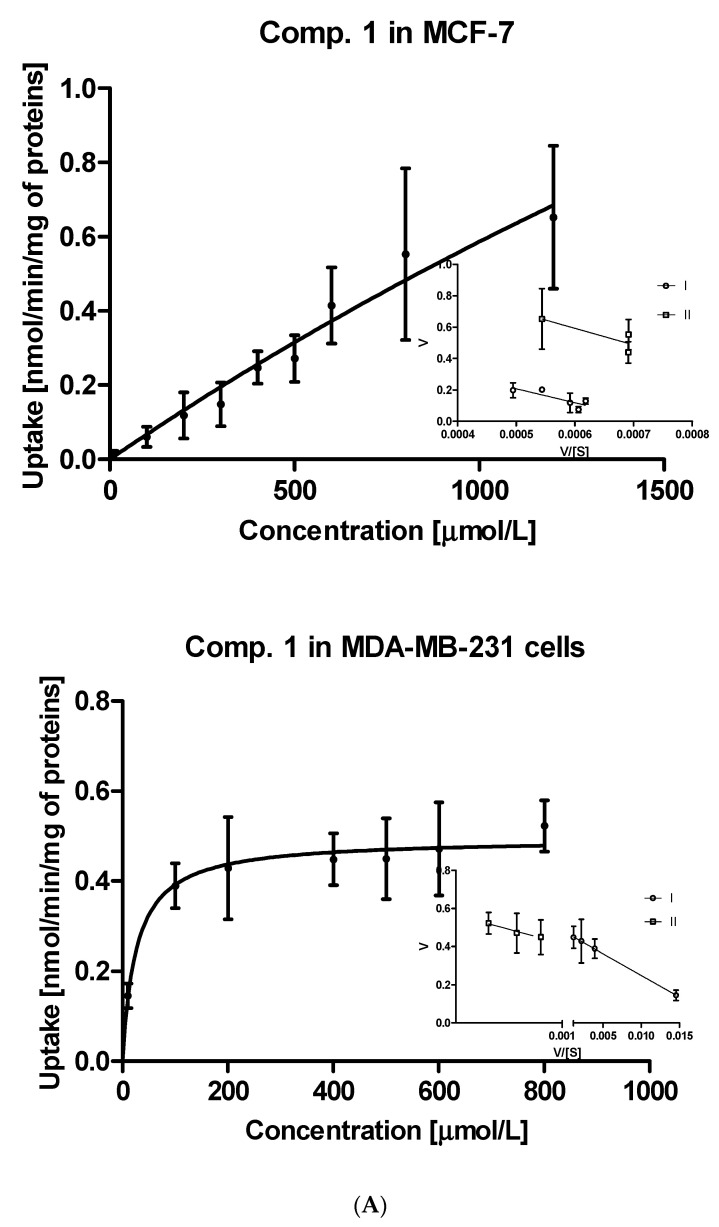

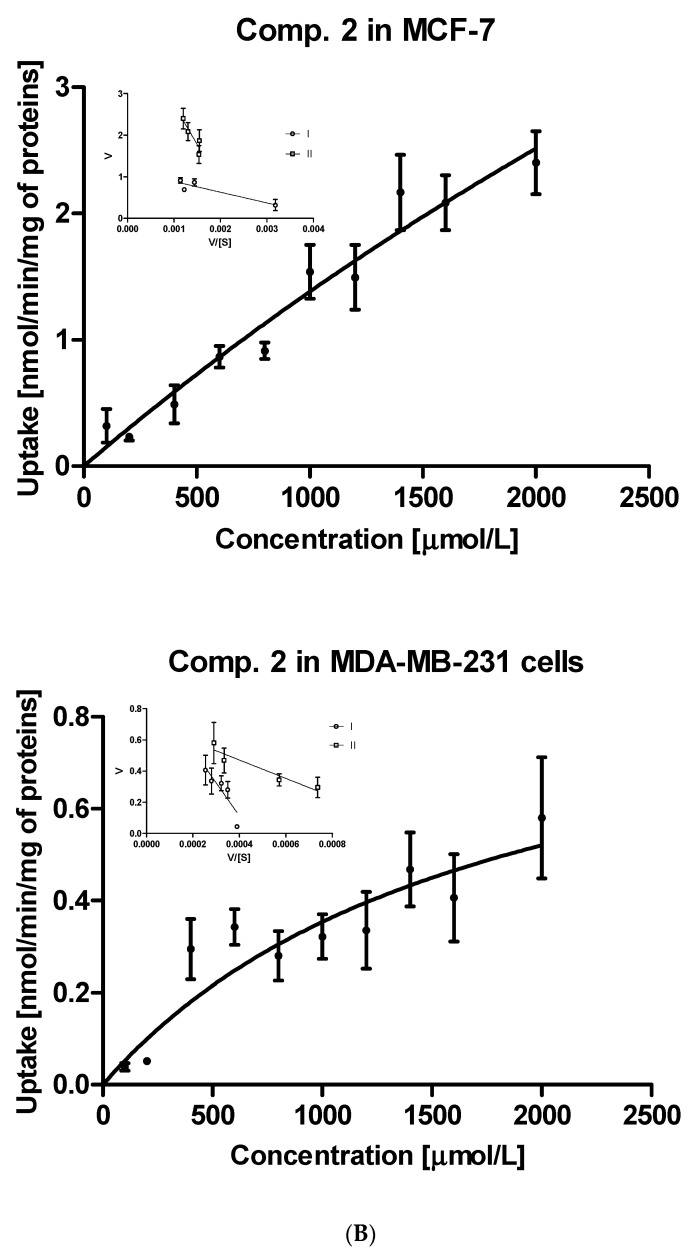
The uptake of selected sulfonamide-based derivatives of metformin (compound **1** (**A**); compound **2** (**B**)) into MCF-7 cells and MDA-MB-231 cells (at the concentrations of 10–2000 µmol/L and Eadie–Hofstee plots for OCTs mediated transport.

Further steps of the intracellular uptake analysis included transformation of the obtained curves into Eadie-Hofstee plots, and subsequent calculation of kinetic parameters of obtained curves (Table S1). Compound **1**, the simplest studied sulfonamide, was transported into MCF-7 and MDA-MB-231 cells with the aid of two transporters differing in the affinity and capacity (marked as I and II, Figure 2A). Both transporters in MCF-7 cells, carrying compound **1** into the cells, appear to have medium affinity for this compound. However, these transporters differ in the capacity since the respective V_max_ values were 0.653 ± 0.124 and 1.225 ± 0.358 nmol/min/mg of protein (Table S1). In turn, both transporters in MDA-MB-231 cells carried derivative **1** into the cells at a low rate (low-capacity transporters) but compound **1** possesses high affinity towards them (low K_m_ values). According to the V_max_/K_m_ ratios of monomethyl derivatives (compounds **2**–**4**) presented in Table S1, both transporters in MCF-7 cells generally appeared to be more efficient than in the case of MDA-MB-231 cells. For instance, compound **2** was transported into MCF-7 cells by two very high-capacity transporters while in the second cell line, compound **2** was uptaken by relatively less efficient transporters (Table S1, Figure 2B). In turn, compound **3** was carried into MCF-7 cells by two transporters presenting comparable affinity and high capacity (high V_max_ values, Table S1). Compound **3** showed a completely different cellular uptake profile in MDA-MB-231 cells (Figure S3, Supplementary information). Based on the arrangement of points on the Eadie-Hofstee plot, we can assume that compound **3** leads to autoactivation of transporter [27]. Both transporters carrying compounds **4** into MCF-7 cells present comparable efficacy (V_max_/K_m_ ratio); however they differ in the affinity and capacity for **4** (Table S1). In contrast, one of the transporters in MDA-MB-231 cells transporting compound **4** is high-affinity low-capacity transporter (K_m_ = 144.6 μmol/L; V_max_ = 0.249 nmol/min/mg proteins). The second transporter behaves more like low-affinity high-capacity transporter (K_m_ = 2930 μmol/L; V_max_ = 1.504 nmol/min/mg proteins). Compound **5** with *p*-propyl chain in the benzene ring presents a similar uptake profile to that of compound **4** in both cell lines. According to the K_m_ and V_max_ values, it appears that in MCF-7 cell line, both transporters participate equally in the transport of compound **5**, while in MDA-MB-231 cells two transporters differ in efficacy, which stems from the uptake profile and fast saturation of transporters (Table S1, Figure S1, Supplementary information).

**Figure 3:**
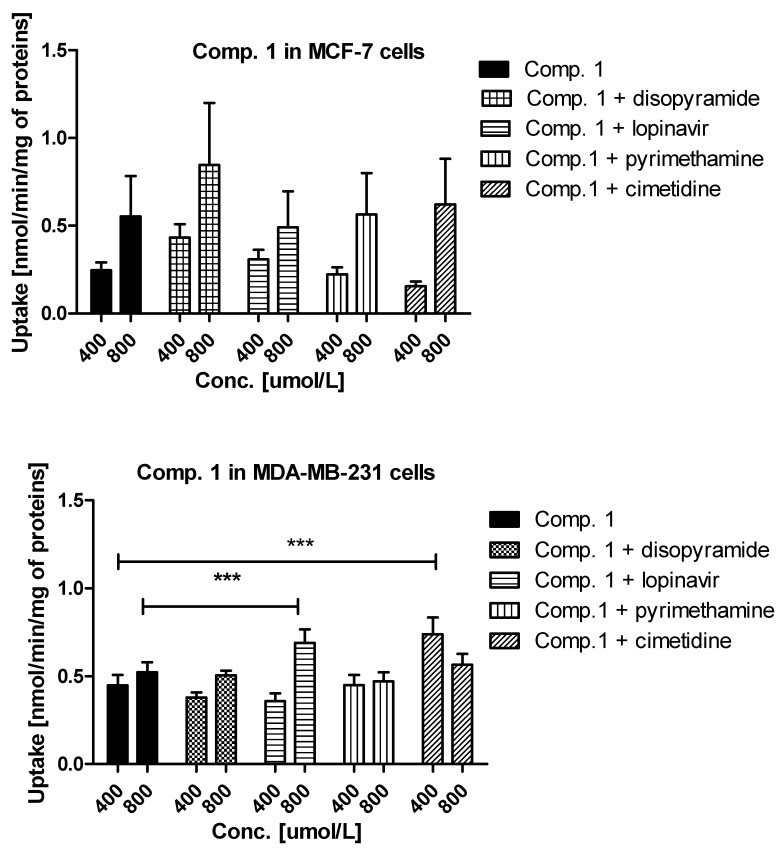

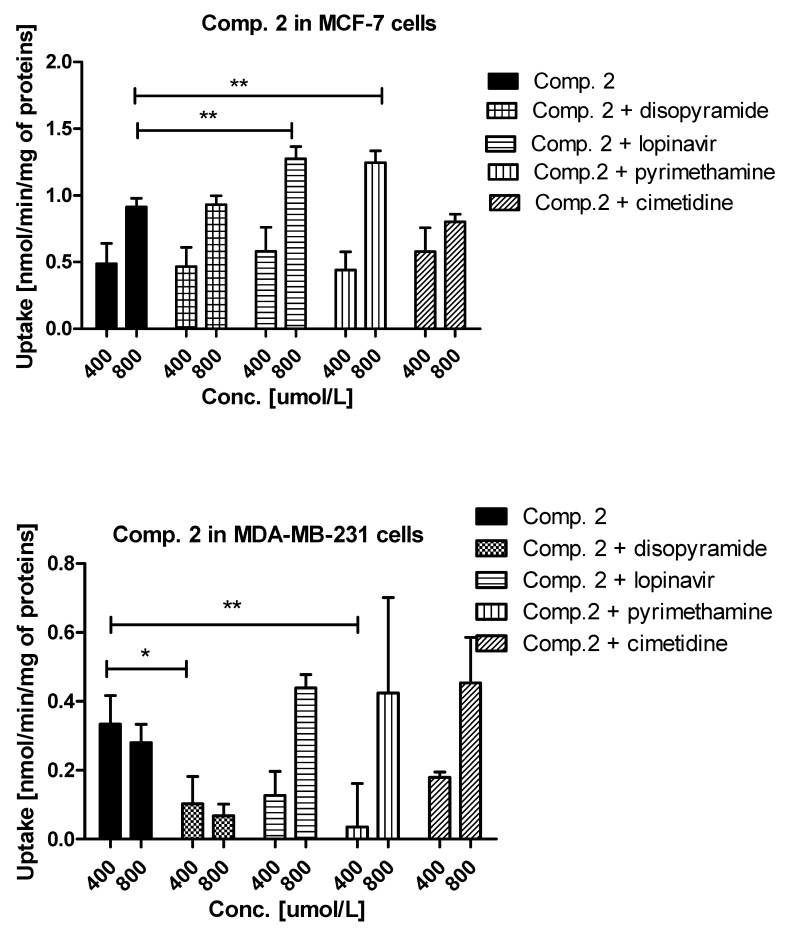
The uptake mechanism of compounds **1** and **2** (400 and 800 µmol/L) into MCF-7 cells and MDA-MB-231 cells. The uptake was determined in the presence of OCT and MATE inhibitors, disopyramide, lopinavir, methenamine, cimetidine (400 and 800 µmol/L) for 10 minutes at 37 °C. The significant differences between pure compounds **1**–**2** and their mixtures with inhibitors are denoted with asterisk. * *p* < 0.05, ** *p* < 0.01, *** *p* < 0.001.

Experiments with OCTs, and MATE inhibitors were performed to explain in detail which transporters are engaged in the cellular uptake of the studied sulfonamides. The effects of selected inhibitors (at 400 and 800 μmol/L) on the uptake of compound **1** are presented in Figure 3. The uptake of compound **1** in MCF-7 cells was decreased, yet insignificantly, only in the presence of cimetidine (0.247 ± 0.044 nmol/min/mg proteins vs. 0.155 ± 0.027 nmol/min/mg proteins, *p* > 0.05), which suggests that OCT3 transporters might participate in the cellular uptake of **1**. In turn, co-treatment of MCF-7 cells with compound **1** and disopyramide resulted in higher uptake of **1** (0.553 ± 0.201 nmol/min/mg proteins vs. 0.846 ± 0.354 nmol/min/mg proteins, *p* > 0.05), which indicates that another medium-affinity transporter is used, when the OCT1 transporter is inhibited. Thus, it can be presumed that disopyramide hinders the studied compound to bind to OCTs and forces them to use other high capacity transporters. With respect to MDA-MB-231 cells, a significantly higher uptake of compound **1** in the presence of both lopinavir and cimetidine was observed (Figure 3). These results suggest that compound **1** is able to utilize some other higher affinity transport mechanism when PMAT or OCT3 are inhibited and that it also has affinity for PMAT. No statistically significant changes in the uptake of **1** in the presence of both disopyramide and pyrimethamine were reported in MDA-MB-231 cells, which implies that OCT1 and MATE1 are not responsible for a moderate uptake of compound **1** in MDA-MB-231 cells.

A relatively high uptake of compound **2** in MCF-7 cells (Figure 3) was profoundly increased in the presence of both lopinavir and pyrimethamine, which means that either another high-capacity transporter is used, when PMAT is inhibited or MATE1, which is normally transporting compound **2** out of the cell as an efflux transporter, is inhibited. In MDA-MB-231 cells, the uptake of compound **2** was significantly inhibited by disopyramide (0.334 ± 0.083 nmol/min/mg proteins vs. 0.103 ± 0.079 nmol/min/mg proteins, *p* < 0.05) and pyrimethamine (0.334 ± 0.083 nmol/min/mg proteins vs. 0.036 ± 0.106 nmol/min/mg proteins, *p* < 0.01). These results reveal that the OCT1 transporter together with MATE1 might be responsible for moderate uptake of compound **2** in MDA-MB-231 cells. No significant differences were observed in the uptake of **3** in the presence of all inhibitors apart from lopinavir for which a significant increase in compound **3** uptake was found (1.508 ± 0.401 nmol/min/mg proteins vs. 2.968 ± 0.789 nmol/min/mg proteins, *p* < 0.001) (Figure S2, Supplementary information). These outcomes suggest a potential interaction with PMAT transporters, which are highly expressed in MCF-7 cells [26]. The low uptake of compound **3** (Figure 1) was inhibited by all studied inhibitors. However, statistically significant results were obtained for lopinavir and pyrimethamine which suggests that PMAT and MATE1 transporters participate in its uptake in MDA-MB-231 cells. The uptake of compound **4** was significantly decreased in the presence of cimetidine (0.540 ± 0.071 nmol/min/mg proteins vs. 0.375 ± 0.049 nmol/min/mg proteins, *p* < 0.01) indicating participation of OCT3 in cellular uptake of **4** in MCF-7 cells. In turn, the uptake of compound **4** in MDA-MB-231 cells was significantly reduced in the presence of pyrimethamine and cimetidine, which suggests that MATE1 and OCT3 might be responsible for its moderate uptake in MDA-MB-231 cells. Importantly, a significant increase in the uptake of **4** in both cell lines was reported in the presence of lopinavir which implies that PMAT transporters participate in its uptake in MCF-7 and MDA-MB-231 cells. Decreased uptake of compound **5** in MCF-7 cells was found in the presence of lopinavir and cimetidine. These findings indicate that compound **5** uses PMAT and OCT3 transporters. On the other hand, the uptake of compound **5** did not change in the presence of disopyramide, lopinavir and cimetidine in MDA-MB-231 cells. A significant increase in the uptake of **5** was reported only at 800 μmol/L of pyrimethamine, so it can be concluded that compound **5** potentially interacts with MATE1 transporters.

Dimethyl derivatives showed differential uptake profiles depending on the position of methyl substituent in the aromatic ring. For instance, in MCF-7 cells both transporters appear to be equally efficient in transport of compound **6**, and could be regarded as medium-affinity (K_m_ = 1260 ± 178.3 and 1077 ± 145.1 μmol/L) high-capacity transporters (V_max_ = 2.713 ± 0.235 and 2.705 ± 0.234 nmol/min/mg of protein, respectively). Substitution change in the aromatic ring into position 2 and 5 (compound **7**) contributed to differential uptake of this compound in MCF-7 and MDA-MB-231 cells. Similarly to the previous compound, compound **7** was uptaken in MCF-7 cells by two high-affinity high-capacity transporters with comparable efficacy (Table S1). In turn, the uptake of compound **7** was much less effective in the case of MDA-MB-231 cells. A completely different cell uptake model was shown for compound **8** which was uptaken more efficiently in MDA-MB-231 cells than in MCF-7 (Table S1, Figures S1 and S3, Supplementary information). This phenomenon was manifested with higher respective V_max_ values, and greater transporter efficacy (V_max_/K_m_ ratios) than in MCF-7 cells. Compound **9** with methyl group in 3 and 5 position in the benzene ring was uptaken in both cell lines at a comparable rate. In MCF-7 cells, both transporters were similarly effective regarding the uptake of **9**, and the can be treated as high-affinity medium-capacity transporters. In MDA-MB-231 cells, differences between both transporters are more pronounced (Table S1). The Eadie-Hofstee analysis of 3,5-dimethyl derivative (compound **9**) uptake showed two transporters with a similar capacity (0.885 ± 0.412 and 1.028 ± 0.463 nmol/min/mg of proteins). However, this derivative seems to possess differential affinity towards the transporters in MDA-MB-231 cells as the respective K_m_ values were different (Table S1). Compound **10** with three methyl groups in the aromatic ring was characterized by considerable uptake in both cell lines (1.794 ± 0.498 nmol/min/mg proteins, and 7.944 ± 0.129 nmol/min/mg proteins, Figures S1 and S3, Supplementary information). The Eadie-Hofstee analysis (Figures S1 and S3) showed a totally different mechanism of uptake of **10** in both cell lines because the reaction rate plotted as a function of the ratio between rate and substrate concentration did not form straight lines. The obtained diagrams resembled more a sigmoidal curve which suggest autoactivation of transporter, and compound induces the activity or amount on the cell membrane of its transporting proteins. We presume that these findings might be associated with a strong, direct effect of this compound on the integrity of cellular membrane. Similar uptake mechanism in MDA-MB-231 cells was also found in the case of compound **6**.

The uptake of dimethyl compounds (**6**–**9**) was also studied in the presence of OCT inhibitors (Figure S2, Supplementary information). In MCF-7 cells, the uptake of compound **6** at lower concentration (400 μmol/L) was increased in the presence of disopyramide; however, at higher concentration (800 μmol/L), the uptake of **6** was significantly reduced (1.026 ± 0.121 nmol/min/mg proteins, and 0.676 ± 0.080 nmol/min/mg proteins). These findings imply that compound **6** utilizes the OCT1 transporter. In the case of MDA-MB-231 cells, a significantly lower uptake of compound **6** was reported in the presence of pyrimethamine (1.165 ± 0.176 nmol/min/mg proteins, and 0.674 ± 0.102 nmol/min/mg proteins, *p* < 0.001). In addition, the uptake of **6** was increased in the presence of disopyramide (1.165 ± 0.176 nmol/min/mg proteins, and 1.647 ± 0.248 nmol/min/mg proteins, *p* < 0.001) which suggest that OCT1 also participates in its uptake. In turn, no significant differences were observed in the uptake of **7** in the presence of all inhibitors (disopyramide, lopinavir, pyrimethamine, and cimetidine) in MCF-7 cells (Figure S2, Supplementary information). However, a slightly decreased uptake of **7** was found in the presence of disopyramide (1.340 ± 0.251 nmol/min/mg proteins, and 0.681 ± 0.127 nmol/min/mg proteins, *p* > 0.05), which suggests that OCT1 may be a medium-affinity high-capacity carrier, responsible for intracellular transport of this compound in MCF-7 cells. A low uptake of compound **7** was inhibited by disopyramide, lopinavir and pyrimethamine suggesting that OCT1 and PMAT might participate in the cellular uptake of **7** in MDA-MB-231 cells. Compound **8** was uptaken efficiently in MCF-7; however, it was much lower than in MDA-MB-231 cells (Figures S1 and S3, Supplementary information). The uptake of this compound at 800 μmol/L in MCF-7 was increased in the presence of all examined inhibitors, while its uptake in MDA-MB-231 was significantly inhibited in the presence of disopyramide and pyrimethamine. The uptake of **8** was also inhibited when co-treated with cimetidine; however, the changes were insignificant (13.102 ± 0.607 nmol/min/mg proteins, and 2.725 ± 2.485 nmol/min/mg proteins, *p* > 0.05) (Figure S2, Supplementary information). These findings suggest that OCT1, OCT3 and MATE1 participate in the cellular uptake of **8** in MDA-MB-231 cells. The uptake of compound **9** in MCF-7 cells showed a similar pattern to that represented by compound **7**, and no significant differences were observed in the uptake of **9** in the presence of inhibitors (Figure S2, Supplementary information). In the case of MDA-MB-231 cells a significantly reduced uptake of compound **9** in the presence of both disopyramide, lopinavir and cimetidine was observed. Therefore, it can be stated that compound **9** uses OCT1, OCT3 and PMAT in the uptake in MDA-MB-231 cells. Results of uptake studies in the presence of inhibitors showed that compound **10** in MCF-7 interacts with the MATE1 transporter since its uptake was increased in the presence of pyrimethamine (1.794 ± 0.499 nmol/min/mg proteins, and 8.295 ± 2.305 nmol/min/mg proteins, *p* < 0.001) (Figure S2, Supplementary information). Compound **10** was transported efficiently in MDA-MB-231 cells, and its uptake was significantly reduced when co-treated with all inhibitors (Figure S2, Supplementary information). This observation might stem from the autoactivation by compound **10** and stronger interaction of inhibitors with transporter.

In summary, the presented results show how chemical modification of the biguanide scaffold into sulfonamides differing with alkyl substituents in the aromatic ring influences the uptake in two unmodified human breast carcinoma cell lines, MCF-7 and MDA-MB-231. These two cell line have been characterized according to the expression of OCT1-3, PMAT and MATE1-2 transporters [19,26]. This is highly important in view of the fact that that expression of OCT transporters correlates with the anti-proliferative and antitumor efficacy of metformin in breast cancer cells [19]. Actually, OCT2 expression in tumor tissue may predict targeted metformin uptake and pharmacological response to the drug [28].

Having analyzed the cellular uptake of the first group of sulfonamides including unsubstituted (compound **1**) or monosubstituted derivatives (**2**–**5**), we can conclude that these compounds show either a comparable uptake rate in both cell lines or they are transported more efficiently in MCF-7 cells (compounds **2** and **3**). These results were further confirmed by the Eadie-Hofstee analysis (Table S1), which revealed two high-capacity transporters participating in compound **2** uptake in MCF-7 cells. A substantially lower uptake of **2** in MDA-MB-231 cells might result from lower affinity towards their transporters, and lower capacity of the transporters expressed as the reduced V_max_/K_m_ ratio. The changes in the uptake of **2** in MCF-7 in the presence of lopinavir indicate interaction with PMAT transporters, which shows greater expression in MCF-7 cells than in MDA-MB-231, and therefore justifies greater uptake of **2** in MCF-7 [19,26]. In turn, in MDA-MB-231 cells the uptake of compound **2** was inhibited by disopyramide which points out that OCT1 transporters participate in its uptake. Weak expression of OCT1 in MDA-MB-231 cells seems to be a good explanation for a poor uptake of compound **2** in this cell line.

**Figure 4:**
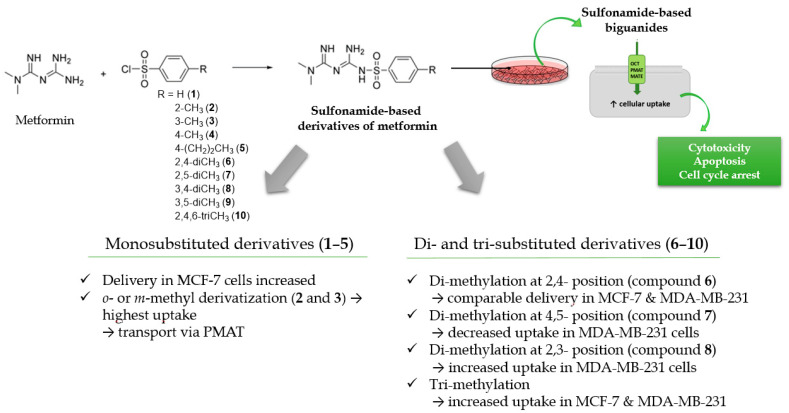
Structure–activity relationship (SAR) analysis of sulfonamide-based metformin derivatives (**1**–**10**).

A more differentiated uptake profile can be observed among di- and tri-substituted compounds (**6**–**10**). By comparing the position of methyl groups in the aromatic ring, we can conclude that the presence of two methyl groups in 2,4- position (compound **6**) results in a comparable cellular uptake in both cells lines (Figure 1), while changing position 4 into 5, results in a subsequent decrease in the uptake in MDA-MB-231 cells (compound **7**). Interestingly, changing the position of the methyl group from 2 to 3 in the aromatic ring (compound **8**) results in a significant increase in the uptake in MDA-MB-231 cells. A comparable uptake of compound **9** with dimethyl substituents in position 3 and 5 confirms this observation. Furthermore, on the basis of the uptake studies in the presence of inhibitors (Table S2, Supplementary information) we suppose that some of the examined sulfonamides are able to use not only OCT transporters but also PMAT and/or MATE in MCF-7 cells and MDA-MB-231 cells. Importantly, since metformin and its derivatives start to utilize a secondary transport mechanisms once the primary transporter is inhibited, it make the analysis of transport mechanism more complex. Another important issue regarding the obtained results is the fact that the studies were conducted using native cancer cells, which can reveal these secondary transport mechanisms, unlike transporter-transfected cells, which cannot mimic more relevant physiological conditions. Summarizing this part of our research, we can indicate the importance of the position and number of substituents in a certain chemical structure (here the biguanide scaffold, Figure 4) during the design and synthesis of new potential anticancer agents, and demonstrate how it affects the efficiency of intracellular transport in cell lines that differ in the expression of transporters.

### 2.2. Cytotoxicity of Compounds **1**–**10**

The next step of our studies was to screen all sulfonamide based derivatives of metformin towards their effects on MCF-7 and MDA-MB-231 viability using WST-1 assay, which proves the mitochondrial function. Results expressed as concentrations inducing a 50% decrease in cell viability (IC_50_) are presented in Table 3.

MCF-7 and MDA-MB-231 cells were treated with sulfonamides **1**–**10** at a concentration range of 1–2000 µmol/L and 10–3500 µmol/L, respectively. Compound **1** without any substituent in the aromatic ring was uptaken in both cell lines in a comparable rate, and demonstrated similar cytotoxic effects in both cell lines (IC_50_ = 1786 ± 123.8 µmol/L in MCF-7 cells, and 1644 ± 122.87 µmol/L in MDA-MB-231 cells). Similar results in a group of monosubstituted compounds, with respect to uptake and IC_50_ values, were also obtained for compounds **4** and **5**. A much different situation can be observed in the case of compounds **2** and **3** which were uptaken more efficiently in MCF-7 cells than MDA-MB-231 cells (Figure 1). These compounds were characterized by much lower IC_50_ values for MCF-7, equaling 87.7 ± 1.18 µmol/L for **2** and 144.6 ± 12.2 µmol/L for **3**.

Adding one methyl group in the benzene ring (compounds **6**–**9**) potentiates cytotoxic effects in MCF-7 cells, which were expressed by substantially lower IC_50_ values with the lowest one reported for compound **8** (IC_50_ = 15.65 ± 1.22 µmol/L in MCF-7 cells). Compound **8** was also found to be the most efficiently transported into MDA-MB-231 cells (13.102 ± 0.607 nmol/min/mg protein), and these results were reflected by the lowest IC_50_ in MDA-MB-231 cells (903.9 ± 115.6 µmol/L). Other dimethyl compounds demonstrated weak cytotoxic properties towards MDA-MB-231 cells which were manifested by high IC_50_ values (Table). The compound possessing three methyl groups in the aromatic ring (**10**) presented high cytotoxic properties in MCF-7 cells (48.46 ± 11.79 µmol/L). It might be expected that relatively low IC_50_ values and profound cytotoxic properties of **10** result from its high uptake in MCF-7, which was approximately 60-fold higher than that of metformin in MCF-7 cells. Compound **10** was also characterized by high uptake in MDA-MB-231 cells (7.944 ± 0.129 nmol/min/mg of proteins), which reflected its effect on viability of MDA-MB-231 (992.1 ± 115.9 µmol/L). However, the fact that this value is approximately 20-fold higher than the one observed with MCF-7 cells should be taken into account. These results suggest that incorporation of two or three methyl groups in the aromatic ring in sulfonamide derivatives enhances the biological effects only in MCF-7 cells independently on the cellular uptake of the compound.

**Figure 5:**
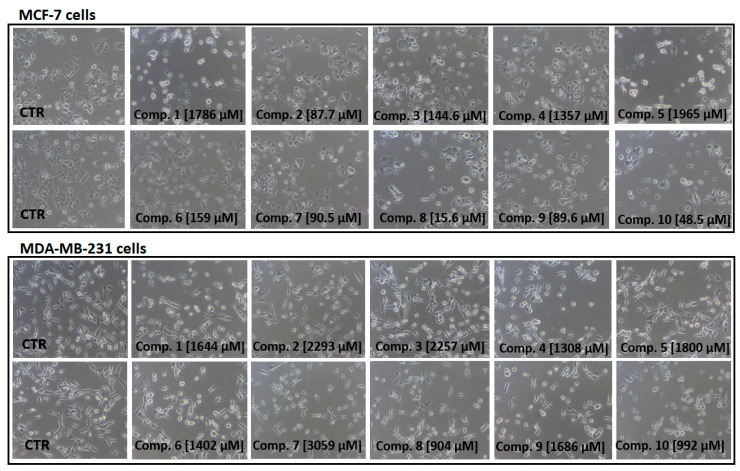
Dose-dependent effect of sulfonamide derivatives of metformin (compounds **1**–**10**) at concentrations corresponding to their IC_50_ values [µmol/L] on MCF-7 and MDA-MB-231 cells viability and morphology after 24 h incubation. Representative cell images are shown (100-fold magnification); CTR–control.

Next, we also examined the effects of all sulfonamides (compounds **1**–**10)** at concentrations ranging from 10 to 3000 µmol/L on the morphology of MCF-7 and MDA-MB-231 cells using light and phase-contrast microscopy. Figure 5 shows the influence of sulfonamides at selected concentration corresponding to the individual IC_50_ value obtained in WST-1 assay. Images in Figure 5 show that all sulfonamides at their IC_50_ concentrations contributed to a significant decrease in the number of viable cells, their density, and alteration in MCF-7 cells morphology. More specifically, a greater number of rounded cells, membrane disruption and inhibition of growth for compounds **1**–**10** were observed. All tested compounds were found to induce comparable changes in MDA-MB-231 cells; however, these changes were observed at higher concentrations (904–3059 µmol/L) compared to MCF-7 cells. For instance, compound **1** at 10–700 µmol/L did not contribute to substantial alterations in the MDA-MB-231 cells morphology. However, membrane disruption, cell shrinkage, rounding, and inhibition of MCF-7 growth were observed at concentrations reflecting IC_50_ value (Figure 5). Similar observations were made for all studied compounds. In this part of studies, a microscopic evaluation of MCF-7 and MDA-MB-231 cells confirms the results obtained in the viability assay since the examined compounds inhibit growth of MCF-7 cells at lower concentrations than MDA-MB-231 cells.

Taking all these results into consideration, we can conclude that chemical modification of the metformin structure into sulfonamides containing a methyl-substituted aromatic ring results in both higher cellular uptake in MCF-7 and MDA-MB-231 cells, and greater biological effects than those of parent drug, metformin. Our previous studies reported that metformin administered in the concentration up to 3000 µmol/L, does not contribute to profound inhibition of MCF-7 and MDA-MB-231 cell growth [25]. These results stem from the low cellular uptake of this drug in both cell lines, which, in turn, weakly express OCT transporters. Moreover, other authors confirm that metformin anti-cancer properties depend on the efficacy of cellular uptake in cancer cell line, and the uptake in turn depends on the expression of the OCT transporters [19,28]. For instance, it was reported that metformin is taken up 13-fold more efficiently in OCT3- BT20 cells compared to unmodified BT- 20 cells [19]. Moreover, our previous studies [26] confirmed that the potency of the cytotoxic activity correlates with the cellular uptake of the compound.

Another important issue is an effect of these novel compounds on the viability of primary cells. Previously, we performed a series of experiments using endothelial cells of human umbilical vein [29]. Reported IC_50_ values were far lower than those presented for MCF-7 cells. For instance, the IC_50_ value of compound **8** was 490 ± 70.1 µmol/L, which is approximately 31-fold lower than in MCF-7 cells (Table 3). The studied compounds were also examined towards interaction with red blood cell membrane [29]. All compounds were found to be hemocompatible since the hemolysis rate after incubation of RBCs with metformin derivatives (**1**–**10**) did not exceed 10% which is regarded as clinically important value. In addition, the effects of compounds **1**–**10** on the morphology of erythrocytes were also carefully monitored. Previously conducted studies [29] showed a small interaction of the examined metformin analogues with the red blood cell membrane since mainly physiological transformation of discocytes into echinocytes and stomatocytes was observed. It means that compounds **1**–**10** do not disturb erythrocyte function. Collectively, the presented modification of the metformin scaffold into benzenesulfonamides with alkyl substituents in the aromatic ring might be considered a promising approach in improvement of cytotoxic properties. The results obtained in cellular uptake studies together with the calculated IC_50_ values in MCF-7 and MDA-MB-231 cells were used to select several compounds for further research. Compounds **2**, **3**, **4**, **8** and **10** were chosen for detailed studies in MCF-7 cells, while compounds **4**, **8** and **10** were further studied in MDA-MB-231 cells.

### 2.3. Apoptosis Assay

Based on the results of uptake studies and cytotoxicity experiments in MCF-7 and MDA-MB-231 cells, we selected five compounds (**2**, **3**, **4**, **8** and **10**) for further studies in MCF-7 cells. Additional studies on MDA-MB-231 cells were also performed for compounds **4**, **8** and **10**. Growth inhibition might be caused by induction of apoptosis in cancer cells. Hence, we decided to stain the cells with Annexin V and propidine iodide, and find out whether selected biguanides with alkyl substituents in the aromatic ring induce apoptosis. Fluorochrome-labeled annexin V is a phospholipid-binding protein, and allows detection of phosphatidylserine (PS) translocated in apoptotic cells using a flow cytometry analysis. In turn, propidine iodide enables to distinguish dead or late-apoptotic cells because it is not permeant to live cells. Studies on apoptosis were also in the focus of our attention due to interesting results regarding the parent drug, metformin. Numerous studies performed on multiple cancer cell lines indicate that metformin may induce apoptosis [30,31,32]. Taking the results of the aforementioned studies and our previous experience into account [33], we assessed the potential of selected biguanides for induction of apoptosis.

Effects of selected compounds on the apoptosis induction in MCF-7 and MDA-MB-231 cells are presented in Table 4 and Table 5 and indicate a significant influence of tested biguanides on programmed cell death.

**Figure 6:**
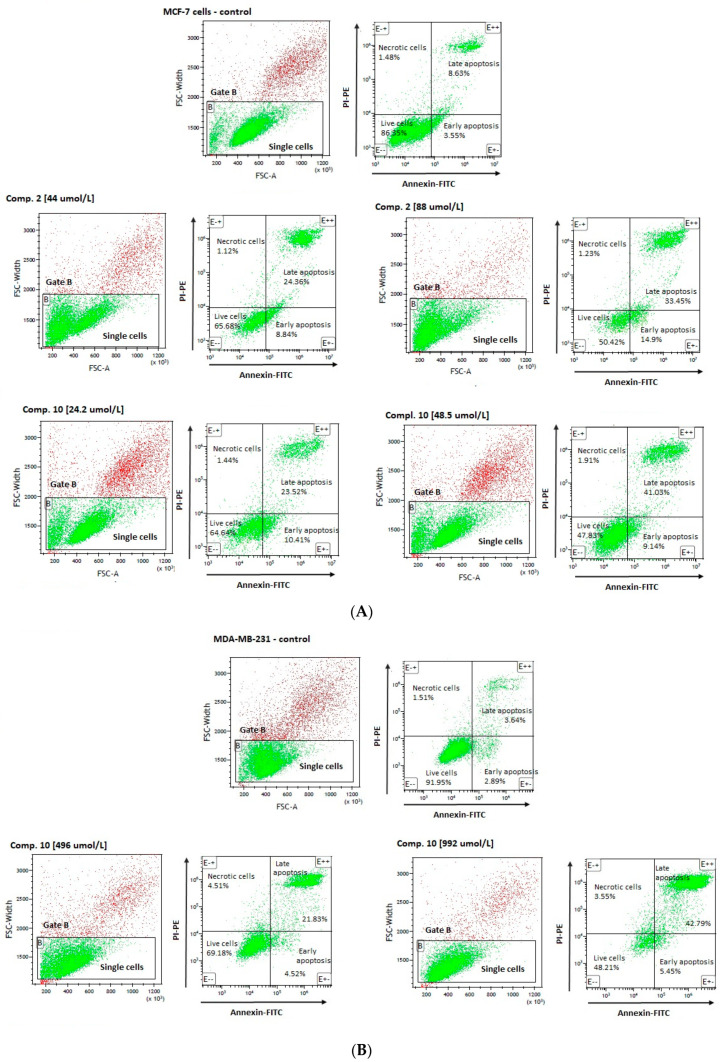
Effects of selected compounds on MCF-7 (**A**), and MDA-MB-231 (**B**) cells viability and apoptosis. (**A**) The cytograms in the upper line are representatives for control MCF-7 cells, the cytogram on the left side—A forward scatter width (FSC-W) vs. forward scatter area (FSC-A) of the analyzed cells. Based on FSC-A parameters, single cells were divided (in the lower part of cytogram), and marked with gate B. The single cells in gate B were analyzed for staining with Annexin V and propidine iodide. Cytograms on the right side—annexin V (*x*-axis) vs. propidium iodide (*y*-axis) plots from the gated cells B show populations corresponding to living cells (annexin V (−) and PI (−)) (E − −)—the lower-left square; early apoptotic cells (annexin V (+) and PI (−)) (E + −) the lower right square, late-apoptotic cells (annexin V(+) and PI (+)) (E + +) the upper right square, and necrotic cells (Annexin V (−) and PI (+)) (E − +) the upper left square. Cytograms in the lower line are representatives for compound **2** at 44 and 88 µmol/L, and compound **10** at 24.2 µmol/L and 48 µmol/L. (**B**) The cytograms in the upper line are representatives for control MDA-MB-231 cells, the cytogram on the left side—A forward scatter width (FSC-W) vs. forward scatter area (FSC-A) of the analyzed cells. Based on FSC-A parameters, single cells were divided (in the lower part of cytogram), and marked with gate B. The single cells in gate B were analyzed for staining with Annexin V and propidine iodide. Cytograms on the right side—annexin V (*x*-axis) vs. propidium iodide (*y*-axis) plots from the gated cells B show populations corresponding to living cells (annexin V (−) and PI (−)) (E − −)—the lower-left square; early apoptotic cells (annexin V (+) and PI (−)) (E + −) the lower right square, late-apoptotic cells (annexin V(+) and PI (+)) (E + +) the upper right square, and necrotic cells (Annexin V (−) and PI (+)) (E − +) the upper left square. Cytograms in the lower line are representatives for compound **10** at 496 µmol/L and 992 µmol/L.

An analysis of MCF-7 cells, stained with annexin V and propidine iodide, revealed a significant decrease in the percentage of living cells (AV − PI −) for all tested compounds (Table 4). These effects were found to depend on the compounds’ concentrations. For example, compound **2** at 44 µmol/L corresponding to its ½ × IC_50_ value significantly decreased the number of viable cells up to 67.49 ± 4.12% in comparison to control samples (83.57 ± 3.30); *p* < 0.001. Compound **2** at higher concentration, equaling its IC_50_ value (88 µmol/L), contributed to a greater decrease in the cellular viability (51.39 ± 4.15%, *p* < 0.001). Importantly, 24-h incubation with compound **2** at 44 µmol/L and 88 µmol/L resulted in a significantly higher percentage of early apoptotic cells (AV + PI −) (9.95 ± 3.93% and 10.04 ± 4.90% vs. 5.29 ± 1.13% for control; *p* < 0.05). Simultaneously, compound **2** induced an increase in the percentage of late-apoptotic cells (AV + PI +) as compared to the control cells. These results also showed dependence on the compound dose (20.74 ± 6.80% for 44 µmol/L (*p* < 0.05), and 37.38 ± 8.36% for 88 µmol/L, (*p* < 0.001)). Representative cytograms showing the effects of compound **2** at 44 µmol/L and 88 µmol/L on apoptosis induction are presented in Figure 6. Co-treatment of MCF-7 cells with selected sulfonamides does not increase the percentage of necrotic cells (AV − PI +); however, a statistically significant increase in this parameter has been observed only in the case of compound **4** at 1357 µmol/L (*p* < 0.05) (Table 4).

A flow cytometric analysis of MDA-MB-231 cells, stained with annexin V and propidine iodide, showed comparable effects of compounds **4**, **8** and **10** regarding induction of apoptosis. However, the observed effects were reported at higher concentrations than those in MCF-7 cells. For instance, stimulation of the cells with derivative **10** at a concentration of 992 μmol/L decreased the viability of MDA-MB-231 cells up to 50.44 ± 4.35% vs. 86.20 ± 5.45% for control, *p* < 0.01). Simultaneously, compound **10** contributed to a significant increase in the percentage of necrotic cells (3.65 ± 0.39% vs. 1.92 ± 0.60%), early apoptotic cells (5.01 ± 0.42% vs. 6.12 ± 2.94%), and late-apoptotic cells (40.90 ± 4.04% vs. 5.75 ± 2.375%). Besides, compounds **4** and **8** were found to induce similar changes in the percentage of early- and late-apoptotic cells (Table 5).

In summary, outcomes of apoptosis experiments reveal that the cytotoxicity of compounds **2**, **3**, **4**, **8** and **10** might stem from their ability to induce apoptosis. Importantly, the examined biguanides are more effective in MCF-7 cells at relatively lower concentrations than in MDA-MB-231 cells which supports the hypothesis that efficient uptake of the examined compounds in MCF-7 cells corresponds positively to their biological effects, including cell growth inhibition, and apoptosis induction.

### 2.4. Cell Cycle Arrest

Since metformin was found to induce cell cycle arrest in the G1 phase in numerous cancer cells [34,35,36] we decided to investigate potential mechanisms of cytotoxic properties of selected biguanides with respect to cell cycle arrest. These experiments were conducted using PI stained cells, and separation of cells in the G0/1, S and G2/M phase was analyzed using a flow cytometer. Results of these studies are shown in Table 6.

**Figure 7:**
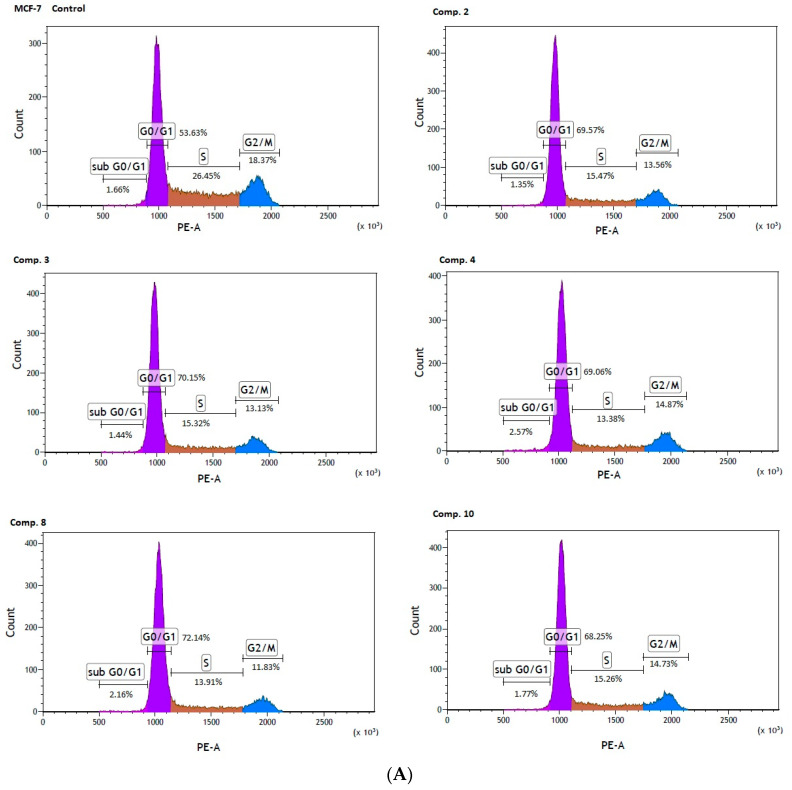

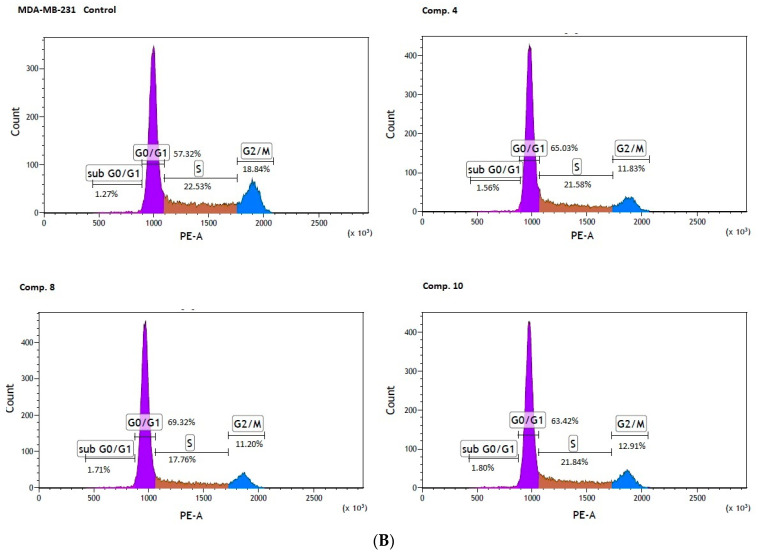
Effect of biguanides on MCF-7 (**A**) and MDA-MB-231 (**B**) cell cycle progression. The cells were co-stimulated with selected compounds at concentration corresponding to IC_50_ values for 24 h. DNA content was determined using a flow cytometry analysis of PI stained cells. Representative cytograms of the cell cycle distribution at G0/1, S and G2/M phases are shown.

Co-stimulation of MCF-7 cells with selected biguanides (**2**, **3**, **4**, **8**, **10**) showed significant changes in the individual phases of the cell cycle. For instance, compound **2** contributed to a significant increase in cells arrested at the G0/G1 phase (69.69 ± 0.94% versus 52.30 ± 1.84% for control, *p* < 0.001). These changes were accompanied by a significant decrease in the S phase (15.42 ± 0.71 versus 26.01 ± 0.70% for control, *p* < 0.001), and the G2/M phase (13.28 ± 0.38% versus 20.45 ± 1.30% for control, *p* < 0.001). The same profile of changes in the cell cycle was observed for the other compounds (Table 6). Representative profiles of the cell cycle distribution of MCF-7 cells stimulated with biguanides **2**, **3**, **4**, **8** and **10**, are depicted in Figure 7A.

Significant changes in cell cycle arrest were also reported in the case of MDA-MB-231 cells. An analysis of the cell cycle distribution showed a significant increase in the percentage of cells at the sub G0/G1 (1.83 ± 0.21% versus 1.40 ± 0.14 for control, *p* < 0.05), and the G0/G1 phase (70.32 ± 1.95% versus 57.09 ± 0.91% for control, *p* < 0.01) in MDA-MB-231 cells treated with compound **8**. Simultaneously, a significant reduction in the percentage of cells arrested in the S, and G2/M fraction were observed (Table 6, Figure 7B). These results are of extreme importance given the fact that cells in the S phase synthesize DNA and have DNA content between 2N and 4N, while in the G2 phase, the cell is prepared for the M phase, during which the cell divides into two separate daughter cells.

In summary, the studied compounds are involved in the G0/G1 arrest of MCF-7 and MDA-MB-231 cells which is a highly demanded characteristic supporting anti-cancer properties of biguanides. Since compounds suppressing cancer cell growth by inducing cell cycle arrest are highly desired agents in therapy [37] the examined biguanides represent a promising strategy for cancer treatment.

### 2.5. Intracellular ROS Generation

**Figure 8:**
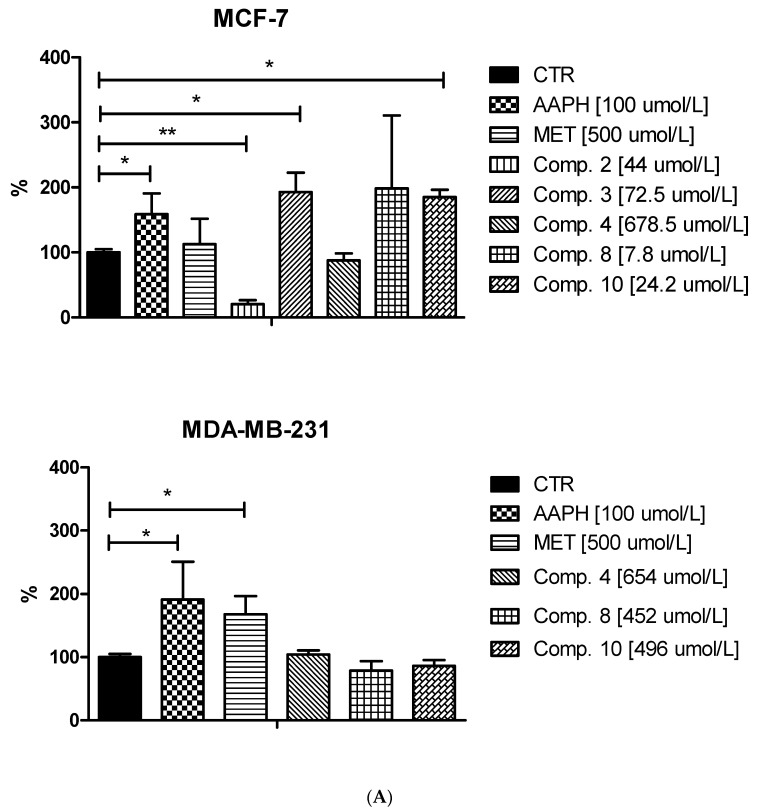

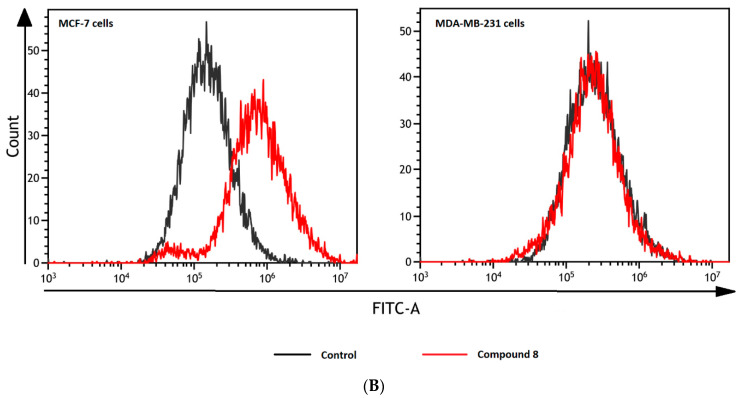
Effects of selected biguanides on the production of intracellular reactive oxygen species in MVF-7 and MDA-MB-231 cells. The experiments were performed using 2′,7′-dichlorodihydrofluorescein diacetate (H_2_DCFDA), a fluorescent indicator of ROS in cells. The fluorescence of stained MCF-7 and MDA-MB-231 cells was measured by flow cytometry. (**A**) Median intensity of fluorescence of 10,000 cells. Results are presented as a percentage value of control samples which constituted 100% (mean ± standard deviation, *n* = 3). The asterisk denotes a statistically significant difference in comparison to control, * *p* < 0.05, ** *p* < 0.01. 2,2’-Azobis(2-amidinopropane) dihydrochloride (AAPH) was used as a positive control. (**B**) Exemplary cytograms of MCF-7 and MDA-MB-231 cells stained with H_2_DCFDA. Black line–control sample, red line compound **8** at concentrations corresponding to ½ IC_50_ value. Compound **8** in MCF-7 cells contributed to increased fluorescence compared to control cells.

Since the unstrained rise in intracellular ROS (reactive oxygen species) levels can contribute to cell cycle arrest, apoptosis and subsequently cell death of tumor cells [38] we conducted research on the influence of selected sulfonamides on the intracellular generation of ROS in MCF-7 and MDA-MB-231 cells. The experiments were performed using 2′,7′- dichlorodihydrofluorescein diacetate (H_2_DCF-DA), which is cell permeable until cleaved by intracellular enzymes forming the anion, H_2_DCF^−^. In turn, this anion is prone to oxidation to DCF which is highly fluorescent [39]. Effects of the tested biguanides together with metformin as a reference drug on the ROS generation in both cell lines is summarized in Figure 8 and Table S3 (Supplementary information). The studies were complemented by experiments using AAPH, a water-soluble azo compound used extensively as a free radical generator. These samples were treated as positive control. Metformin, the parent drug of the studied compounds, was found not to affect ROS generation in MCF-7 cells (226,427.2 ± 60,332.4 RFU vs. 204,422.3 ± 17,741.1 for control; *p* > 0.05). This observation was similar to results obtained by Ariaans et al. [40]. Interestingly, in MDA-MB-231 cells, metformin contributed to a significant increase in the ROS level (305,732.8 ± 36,879.5 RFU vs. 184,715.4 ± 23,171.9 for control, *p* < 0.05). Metformin (>1 mM) was also found to induce ROS production in HeLa cells, which may reflect disruption of the mitochondrial function [41].

Treatment of MCF-7 cells with most of the studied compounds resulted in an excessive increase in ROS generation. For instance, compound **3** at 72.5 μmol/L contributed to 1.91-fold increase in the ROS level (*p* < 0.05; Figure 8). One exception in this group of biguanides is compound **2**, which statistically caused approximately 80% reduction in the amount of generated ROS compared to the control samples. In the case of MDA-MB-231 cells, we did not observe such substantial effects on intracellular ROS generation. Despite increased fluorescence reported after stimulation of the cells with biguanides **4** and **8**, the results were not statistically significant.

In summary, a considerable increase in intracellular ROS generation was observed mainly in the case of MCF-7 cells treated with sulfonamides **3**, **8** and **10**. Based on the results published by Hsieh Li et al. [41], we presume that those biguanides are able to disrupt the electron transport chain and destroy the mitochondrial membrane potential, which may result in increased ROS levels. This in turn can be partially responsible for the observed cell cycle arrest, or apoptosis in MCF-7 cells, similarly to clinically used cancer chemotherapy [38].

### 2.6. Migration Test

**Figure 9:**
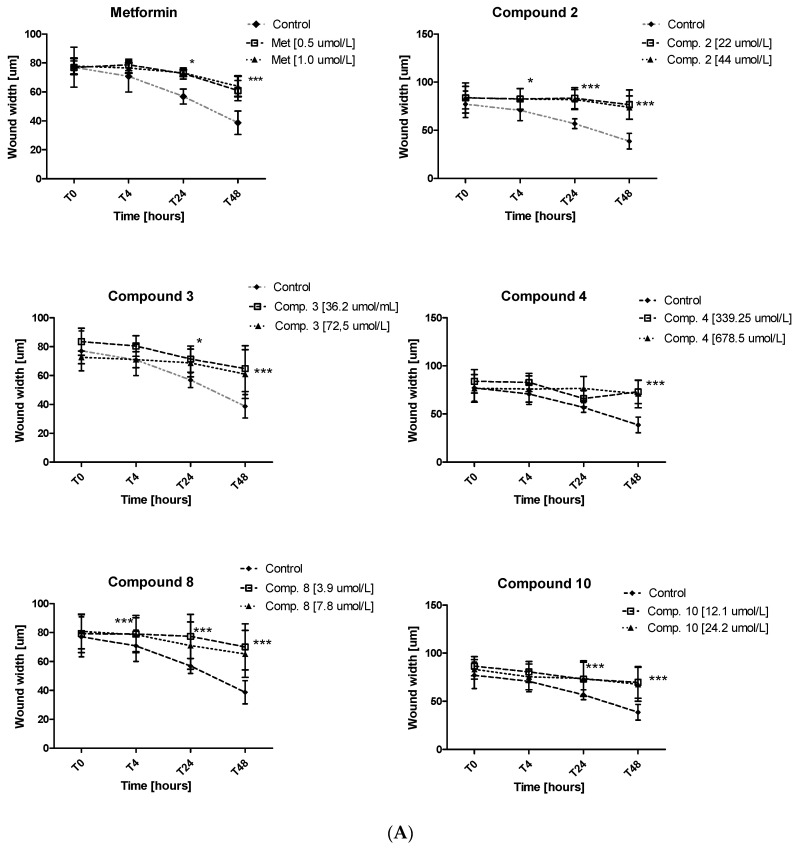

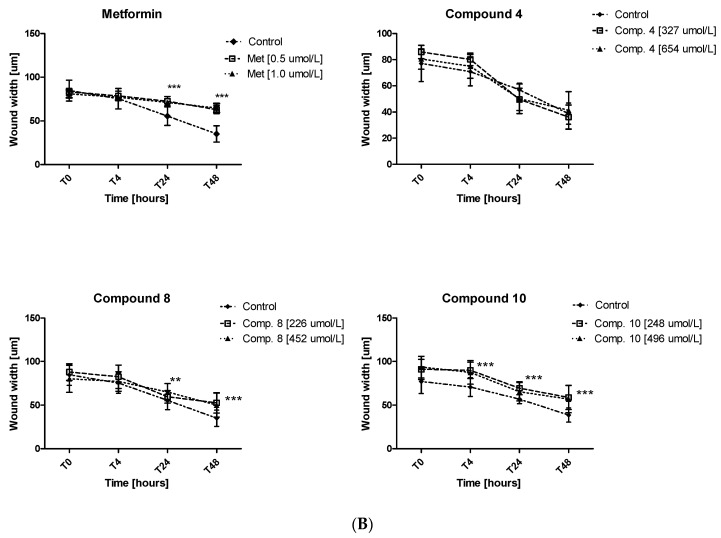
Inhibition of cell migration in the presence of metformin and its sulfonamide derivatives. MCF-7 (**A**) and MDA-MB-231 (**B**) cell migration was evaluated using wound healing assay. Graphs depict changes of the wound width [µm] during 48 h in the absence (control) and in the presence of the examined compounds at various concentrations. Results are presented as the mean ± SD (*n* = 8–12). Asterisks denote statistically significant changes between the examined compounds and control *p < 0.05; ** *p* < 0.01; *** *p* < 0.001.

Excessive ability of cancer cells to migration plays a significant role in cancer invasion and metastasis. Herein, the potential of metformin and its sulfonamide derivatives to affect MCF-7 and MDA-MB-231 cell migration was determined using in vitro wound healing assay. After causing a wound, both cell lines were exposed to different concentrations of the studied compounds for 48 h. The ability of biguanides of affecting MCF-7 and MDA-MB-231 cell migration was inspected microscopically after 4, 24 and 48 h of treatment. The potential of attenuating MCF-7 cell migration of compounds **2**, **3**, **4**, **8** and **10** as well as the potential of attenuating the migration of MDA-MB-231 cells by compounds **4**, **8**, **10** are depicted in Figure 9. Numerical data are presented in Tables S4 and S5 (Supplementary information). Metformin at 500 and 1000 µmol/L was found to substantially affect MCF-7 cell migration after 24 and 48 h of co-treatment. For instance, 48-h incubation with metformin (1000 µmol/L) resulted in a greater wound width in comparison to unstimulated cells (73.11 ± 2.28 µm vs. 56.84 ± 5.13 µm for control, *p* < 0.05). These results are in line with other data which show that metformin inhibits cancer invasion and metastasis, and therefore can improve prognosis of cancer patients treated with metformin [42].

Furthermore, other tested compounds were characterized by significant properties towards inhibition of MCF-7 cell migration which were manifested by an increased width of the wound in comparison with control (Figure 9A). Figure S4 (Supplementary information) shows representative images of wound closure at 0, 4, 24 and 48 h of stimulation with selected compounds in MCF-7 and MDA-MB-231. The migration test also showed that compounds **8** and **10** significantly inhibited MDA-MB-231 cell migration, while compound **4** did not affect the wound width over the entire period of time. The greatest effect on the wound width was observed after 48 h of incubation (Table S5, Figure 9B). After 48 h of testing, approximately 58.7% of wound closure in control cells was observed, while metformin and compounds **8** and **10** demonstrated a significantly decreased ability to fill the damage area. For instance, compound **10** prevented MDA-MB-231 cell migration at both tested concentrations, starting with hour 4 of incubation (*p* < 0.01) (Figure 9B). These results prove beneficial properties of the tested compounds, particularly when we consider the fact that cancer invasion and metastasis transform regionally localized tumors into a systemic and lethal disease.

## 3. Materials and Methods

### 3.1. Breast Cancer Cell Culturing

MCF-7 human breast adenocarcinoma cells (HTB-22) and MDA-MB-231 human breast adenocarcinoma cells were obtained from the American Type Culture Collection (ATCC, Manassas, VA, USA), and Sigma Aldrich (European Collection of Authenticated Cell Cultures (ECACC, Public Health England, Salisbury, UK)), respectively. These cell lines were selected on the basis of the differences of OCT and PMAT transporters expression [26].

Both cell lines were cultured in standard conditions (37 °C, 5% CO_2_) using Dulbecco’s modified Eagle medium (DMEM, Gibco, Thermo Fisher Scientific, Waltham, MA, USA) supplemented with L-glutamine (2 mM, Gibco, Thermo Fisher Scientific, Waltham, MA, USA), heat-inactivated fetal bovine serum (10%, Gibco, Thermo Fisher Scientific, Waltham, MA, USA), penicillin (50 U/mL, Gibco, Thermo Fisher Scientific, Waltham, MA, USA), and streptomycin (50 μg/mL, Gibco, Thermo Fisher Scientific, Waltham, MA, USA). Once the cells reached 80% confluence in culture bottles (75 cm^2^), the cells were washed twice with DPBS solution (Gibco, Thermo Fisher Scientific, Waltham, MA, USA), and harvested using 5 mL of accutase (Sigma Aldrich, St. Louis, MO, USA). Cells of passages between 8–15 were used in the experiments.

### 3.2. Studied Compounds

The structure of the examined sulfonamide derivatives of metformin (compounds **1**–**10**) are depicted in Table 1. The chemical characterization and basic properties were described elsewhere [29]. The analyzed compounds **1**–**10** were also reported to be stable in 0.1 M NaOH and TBS buffer in the abovementioned study.

### 3.3. Uptake Studies of Metformin Derivatives

The cellular uptake of metformin derivatives (**1**–**10**) in MCF-7 and MDA-MB-231 cells was performed in 24-well plates by incubation of the cells seeded at the density of 1 × 10^5^ cells with 10–3000 μmol/L of compounds in 250 μL of pre-warmed HBSS buffer (Hank’s Balanced Salt Solution) at 37 °C for 10 min (linear part of the cellular uptake). These concentrations were chosen based on the therapeutic concentrations of metformin (lower concentrations) and the most frequently tested concentrations in in vitro studies (>100 µmol/L) [43]. The content of HBSS buffer was previously published [25]. Afterwards, the cells were washed three times with 500 μL of ice-cold HBSS and lysed with 0.1 M NaOH (250 μL). The solutions of all wells were collected, centrifuged (5 min, 1400 rpm) and the supernatants were analyzed by the HPLC method. The concentration of each compound in the cells was calculated on the basis of the standard curve prepared by spiking known amounts of every compound on the cells layer in 250 μL of 0.1 M NaOH.

The concentration of the examined compounds (**1**–**10**) in cells lysates was assessed using the high-performance liquid chromatography (HPLC) system, consisting of an Agilent 1100 binary pump (Agilent Technologies Inc., Wilmington, DE, USA), a 1100 micro vacuum degasser, an HP 1050 Autosampler, an HP 1050 variable wavelength detector (operated at 235 nm). The chromatographic separations of compounds **1**–**10** were conducted on an Agilent Zorbax SB-C18 analytical column (4.6 mm × 150 mm, 5 μm) (Agilent Technologies Inc., Wilmington, DE, USA) using isocratic elution of water containing 0.1% formic acid (pH ca. 3.0) and acetonitrile containing 0.1% formic acid with a changing ratio of 75:25 (*v*/*v*), depending on the compound. The analyses were conducted at room temperature (RT). The detailed procedure of chromatographic analysis was described in our previous paper [29]. The studies were performed in quadruplicates.

The protein concentrations in representative wells of each plate were assessed using Bio-Rad protein assay, according to the Bradford method (EnVision, PerkinElmer, Inc., Waltham, MA, USA).

### 3.4. Uptake of Metformin Derivatives in the Presence of OCT and MATE Inhibitors

The uptake of sulfonamide-based derivatives of metformin (**1**–**10**) in MCF-7 and MDA-MB-231 cells was studied in the presence of disopyramide, cimetidine (OCT1 and OCT3 inhibitors), lopinavir (OCT and PMAT inhibitor) and pyrimethamine (MATE, and OCT2 inhibitors). The protocol of these experiments was analogic to the basic experiments (Section 3.3). The cells were seeded on 24-well plates, and were incubated (37 °C, 10 min) with the tested compounds at concentrations of 400 and 800 μmol/L and above-mentioned inhibitors in 250 μL of pre-warmed HBSS buffer. The assessment of compound concentration was conducted using the HPLC method.

### 3.5. Cancer Cell Viability and Morphology

The effects of metformin derivatives (**1**–**10**) on the viability of MCF-7 and MDA-MB-231 cells were assessed using colorimetric WST-1 assay (Takara, Takara Bio Europe, Germain-en-Laye, France). The procedure for viability studies was described previously [25]. Briefly, after 24 h incubation at standard conditions, the cells (1 × 10^4^ per well, 96-well plates) were treated with various concentrations of compounds **1**–**10**, diluted in cell culture medium (10 µL + 90 µL) for another 24 h (37 °C, 5% CO_2_). Then, the cells were washed with 100 μL culture medium, and incubated for 1.5 h with WST-1 reagent dissolved in medium (final volume 100 μL). Absorbance measurements were recorded at 450 nm using a microplate reader (iMARK, Bio-Rad, Hercules, CA, USA). The absorbance of control samples (pure medium) constituted 100% viability, while the results of samples treated with test compounds are expressed as a percentage value of the control samples. The data were presented as the mean ± standard deviation (SD), *n* = 8, coefficient of variability (CV) = 2.65–5.94% depending on the compound. Based on the obtained data IC_50_ values were calculated using concentration-response curves (GraphPad Prism 5 Software, San Diego, CA, USA).

The effects of metformin derivatives on the morphology of MCF-7 and MDA-MB-231 cells were examined using an inverted microscope with phase contrast (Opta-Tech, software OptaView 7, Warsaw, Poland). The morphology of cancer cells was assessed after 24-h incubation with compounds **1**–**10** at various concentrations.

### 3.6. Cell Apoptosis Assay

Both cell lines were seeded on 24-well plates (5 × 10^4^ per well) and allowed to adhere overnight under standard conditions (37 °C, 5% CO_2_). The following day the medium was discarded from all wells and replaced with fresh portion of medium alone (control samples) or medium with tested compounds. The cells were incubated for additional 24 h. Afterwards, the cells were harvested, collected to Eppendorff tubes, and washed twice with cold cell staining buffer (Biolegend, London, United Kingdom). The final step of sample preparation included addition of binding buffer to the cell pellets, and staining with propidine iodide (PI; 10 μL) and FITC-Annexin solutions (AV; 5 μL) (FITC Annexin V Apoptosis Detection Kit with PI (Biolegend, London, UK)). The samples were incubated for 20 min at room temperature in the dark, followed by an analysis conducted on a cytometer (CytoFlex, blue laser, 480 nm, Beckman-Coulter, Indianapolis, IN, USA). The analysis was conducted using Kaluza 2.1 (Beckman-Coulter, Indianapolis, IN, USA) software. Annexin V (−) and PI (−) cells were considered living cells, annexin V (+) and PI (−) early-apoptotic cells, annexin V (+) and PI (+) late-apoptotic cells, and annexin V (−) and PI (+) necrotic cells.

The experiments were conducted in replicates (*n* = 3–6), 10,000 cells were analyzed. The variability coefficient for the assay was determined (CV = 2.12–13.31%, depending on the calculated cell population).

### 3.7. Cell Cycle Analysis

MCF-7 and MDA-MB-231 cells were seeded in 6-well plates (2 × 10^5^ cells per well) and cultured for 24 h under standard conditions. Afterwards, the cells were treated with the tested compounds at concentrations corresponding to IC_50_ for 24-h treatment. Then, the cells were collected, transferred to Eppendorf tubes, washed twice with cold PBS (1 mL), followed by fixation in cold 70% ethanol at 4 ºC for 48 h. Prior to the analysis, the cells were centrifuged (3600 rpm, 5 min), and washed twice with PBS. The solution was discarded, the pellets were suspended in 200 µL of PBS, incubated with ribonuclease (Sigma Aldrich, St. Louis, MO, USA) (37 °C, 30 min, 25 µL, final concentration in a sample was 100 µg/mL) and stained with propidine iodide (Sigma Aldrich, St. Louis, MO, USA) (RT, 20 min, 12.5 µL, final concentration in a sample was 50 µg/mL). The final volume of the sample was 250 µL.

DNA content and the number of cells in individual cell cycle phases were measured by flow cytometry (CytoFlex, blue laser, 480 nm, Beckman-Coulter, Indianapolis, IN, USA). The analyses were conducted for representative 20,000 cells of each sample. The percentage of cells in each cell cycle phase was calculated from live cells that were expressed as 100%. The data are presented as the mean ± SD of three independent experiments. The coefficient of variability for the test was calculated (CV = 1.60–9.60%, depending on the cell population).

### 3.8. Intracellular ROS Generation

MCF-7 and MDA-MB-231 cells were cultured according to the procedure described in Section 3.6. After 24-h incubation, the cells were exposed to the tested compounds at concentrations equaling ½ of IC_50_ value of each compound or metformin (500 µmol/L) as reference substance. 2,2’-Azobis(2-amidinopropane) dihydrochloride (AAPH; (Sigma Aldrich, St. Louis, MO, USA) was used as positive control. After 24 h treatment, control and treated cells were collected to Eppendorf tubes, and centrifuged (5 min, 1100 rpm). The cells were washed with cold staining buffer (Biolegend, London, UK) followed by 30 min incubation with 2 µL of H_2_DCFDA (2′,7′-dichlorodihydrofluorescein diacetate; Thermo Fisher Scientific, Waltham, MA, USA) (final concentration 2 µmol/L). The fluorescence of the samples was measured by flow cytometry (CytoFlex, blue laser, 480 nm, Beckman-Coulter, Indianapolis, IN, USA), and the results were analyzed using Kaluza 2.1 (Beckman-Coulter, Indianapolis, IN, USA) software. 10,000 cells were analyzed from each sample (plate well) and the data are presented as the mean ± SD, *n* = 3. The coefficient of variation for the assay was determined (CV = 8.68%, *n* = 3).

### 3.9. Wound Healing Assay

MCF-7 and MDA-MB-231 cells were cultured on 24-well plates (5 × 10^4^ per well) and incubated overnight under standard conditions. Then, the confluent cells were wounded by scratching with 100-μL pipette tip, the wells were rinsed with 250 μL of fresh medium and filled with the same volume of medium containing compounds at various concentrations or pure medium (control samples). The plates were incubated for 48 h at 37 °C (5% CO_2_) during which migration of cells was monitored using an inverted microscope (Opta-Tech, Warsaw, Poland) with 4× objective. The images of cells migration were not thoroughly investigated after addition of compounds (T_0_), after 4-, 24- and 48-h incubation (T_4_, T_24_, T_48_). The images were analyzed by microscope software (Opta-Tech Software, Warsaw, Poland), and the width of the scratch area was measured. The results are presented as the mean ± SD of the scratch width (*n* = 6–9). The variability coefficient for the assay was determined (CV = 3.58–11.54%, *n* = 6, depending on the time point).

### 3.10. Data Analysis

The statistical calculations were performed using a commercially-available package (Statistica 12.0, StatSoft, Kraków, Poland; GraphPad Prism 5, San Diego, CA, USA). The results are presented as the mean ± SD for variables with a normal distribution of values, subsequently verified with the Shapiro-Wilk test. Statistical differences between the groups were tested using one-way ANOVA, followed by a subsequent post hoc test (Dunnett’s or Tukey’s test). Variables with non-normal distributions were compared using the Wilcoxon signed rank test. Results of all the tests were considered significant at *p*-values lower than 0.05.

The uptake of metformin derivatives in MCF-7 and MDA-MB-231 cells was analyzed in relation to the concentration of compounds and the obtained curves were then transformed into Eadie-Hofstee plots and analyzed to calculate K_m_ and V_max_ values. The concentration contributing to the 50% of cell growth inhibition (IC_50_ values) were calculated using the nonlinear regression analysis (fitting the curve to log (concentration) vs. cellular response).

## 4. Conclusions

In conclusion, in this study we demonstrated how chemical transformation of the biguanide scaffold into sulfonamides with alkyl substituents in the aromatic ring improves OCT mediated cellular uptake in two human cancer cell lines and how it affects the biological activity of these molecules. Ten sulfonamide metformin derivatives that differ in a number and position of alkyl substituents in the benzene ring (compounds **1**–**10**) were comprehensively studied in two unmodified breast cancer cell lines, MCF-7 and MDA-MB-231 differing with the expression of OCT1-3 transporters on their surface. In general, all the examined biguanides were characterized by a greater cellular uptake in both MCF-7 and MDA-MB-231 cells, and more profound biological activity than those of the parent drug, metformin.

The most effective cellular uptake in MCF-7 cells among unsubstituted or monosubstituted sulfonamide-based derivatives (**1**–**5**) was reported for compounds with methyl substituent in *ortho*- and *meta*- position in the benzene ring (compounds **2** and **3**). An analysis of kinetic parameters of the uptake profiles showed two high-capacity transporters participating in the uptake of both the compounds in MCF-7 cells. Effective intracellular transport of these compounds in MCF-7 cells was accompanied by high cytotoxic activity expressed as low IC_50_ values (87.7 ± 1.18 µmol/L for **2** and 144.6 ± 12.2 µmol/L for **3**). Addition of one or two methyl groups in the benzene ring (compounds **6**–**10**) resulted in improvement of cytotoxic effects in MCF-7 cells which were manifested by substantially lower IC_50_ values with the lowest one reported for compound **8** (IC_50_ = 15.65 ± 1.22 µmol/L in MCF-7 cells). Compound **8** was also efficiently transported into MDA-MB-231 cells (13.102 ± 0.607 nmol/min/mg protein), which resulted in the lowest IC_50_ in MDA-MB-231 cells (903.9 ± 115.6 µmol/L). These results show the importance of the number and position of alkyl substituents in the aromatic ring in relation to the efficacy of intracellular transport and cytotoxic properties of sulfonamide-based biguanides.

Cytotoxicity of sulfonamide-based derivatives of metformin **2**, **3**, **4**, **8** and **10** partially results from their ability to induce early and late apoptosis. Furthermore, the studied compounds were found to arrest cell cycle in the G0/G1 phase. Increased intracellular ROS generation after co-treatment with the examined compounds suggests that they disrupt the electron transport chain and alter mitochondrial respiration. Finally, the studied biguanides were also found to inhibit cancer cell migration, which proves their multidirectional mechanism of action. Importantly, the tested biguanides are more effective in MCF-7 cells, including apoptosis induction and cell cycle arrest, at relatively lower concentrations than in MDA-MB-231 cells.

Collectively, these data suggest that a substantial variability in expression of OCT and PMAT transporters among cancer cell lines may contribute to changes in the cellular uptake of the drug and limit its cytotoxic effects. Results of these studies emphasize the extremely important role of transporters in the design and synthesis of novel potential anti-cancer agents.

## Figures and Tables

**Table 1:** Structure of examined compounds and their basic properties.

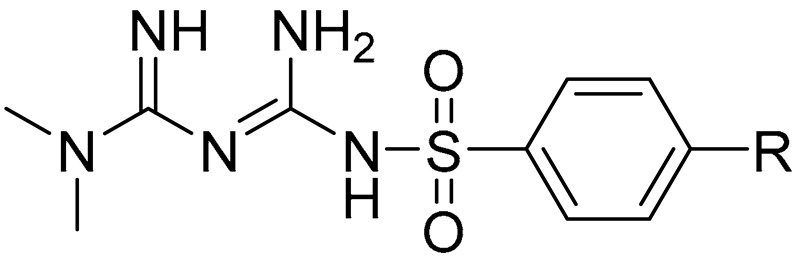			
Compound	R Group	*M*w (g/mol)	cLogD ^a^
**Metformin**	-	129.17	−1.32
**1**	H	269.32	0.80
**2**	2-CH_3_	283.35	1.30
**3**	3-CH_3_	283.35	1.30
**4**	4-CH_3_	283.35	1.30
**5**	4-(CH_2_)_2_CH_3_	297.38	1.47
**6**	2,4-diCH_3_	297.38	1.80
**7**	2,5-diCH_3_	297.38	1.80
**8**	3,4-diCH_3_	297.38	1.75
**9**	3,5-diCH_3_	297.38	1.80
**10**	2,4,6-triCH_3_	311.40	2.30

^a^ Calculated by ChemDraw Professional (v. 16.0.1.4(77), Perkin Elmer Informatics, Inc., Waltham, MA, USA).

**Table 2:** K_m_ and V_max_ values for the uptake of sulfonamide derivatives **1**–**10** in MCF-7 and MDA-MB-231 cells (Michaelis Menten curves).

	Kinetic Parameters of Sulfonamide Uptake					
	MCF-7 Cells			MDA-231 Cells		
Compound	K_m_ [μmol/L]	V_max_ [nmol/min/mg]	V_max_/K_m_	K_m_ [μmol/L]	V_max_ [nmol/min/mg]	V_max_/K_m_
**Metformin**	5583 ± 1560 ^#^	0.801 ± 0.296 ^#^	0.00014 ^#^	3375.0 ± 952 ^#^	0.718 ± 0.181 ^#^	0.000212 ^#^
**1**	6170 ± 778	4.206 ± 2.666	0.00068	26.21 ± 8.798	0.4946 ± 0.021	0.01887
**2**	9070 ± 3913	13.92 ± 6.151	0.00153	1784 ± 714.1	0.985 ± 0.293	0.00055
**3**	312.9 ± 116.7	1.684 ± 0.168	0.00538	NE	NE	NE
**4**	NE	NE	NE	716.6 ± 187.6	0.563 ± 0.132	0.00078
**5**	4508 ± 1992	3.664 ± 2.056	0.00081	1178 ± 525.3	1.292 ± 0.360	0.00109
**6**	819.5 ± 251.4	2.180 ± 0.399	0.00266	NE	NE	NE
**7**	613.2 ± 202.8	2.291 ± 0.321	0.00374	167.6 ± 91.38	0.225 ± 0.024	0.00134
**8**	3454 ± 1103	5.787 ± 2.520	0.00167	3140 ± 1876	65.66 ± 27.85	0.02091
**9**	2601 ± 849	3.174 ± 1.619	0.00122	NE	NE	NE
**10**	843.6 ± 256.1	3.243 ± 0.593	0.00384	110.6 ± 8.991	8.789 ± 0.119	0.07946

NE—not estimated (linear dependency up to the maximal tested concentrations); ^#^ kinetic parameters of metformin uptake in MCF-7 cells and MDA-MB-231 cells were reported previously [25].

**Table 3:** The effects of metformin derivatives on MCF-7 and MDA-MB-231 cells viability. Results (IC_50_ values, µmol/L) are presented as the mean ± SD (*n* = 8).

Compound	MCF-7 Cells [µmol/L]	MDA-MB-231 Cells [µmol/L]
**1**	1786 ± 123.8	1644 ± 122.87
**2**	87.7 ± 1.18	2293 ± 1261
**3**	144.6 ± 12.2	2257 ± 1095
**4**	1357 ± 119.7	1308 ± 118.7
**5**	1965 ± 136.1	1800 ± 142.42
**6**	159.0 ± 12.34	1402 ± 119.2
**7**	90.49 ± 11.79	3059 ± 1291
**8**	15.65 ± 1.22	903.9 ± 115.6
**9**	89.56 ± 11.7	1686 ± 124.3
**10**	48.46 ± 11.79	992.1 ± 115.9

**Table 4:** Annexin V-FITC/PI double staining analysis of apoptosis in MCF-7 cells.

Compound [μmol/L]	Living Cells [E − −] [%]	Necrotic Cells [E − +] [%]	Early Apoptotic [E + −] [%]	Late Apoptotic [E + +] [%]
Control MCF-7	83.57 ± 3.60	1.92 ± 0.67	5.29 ± 1.13	9.23 ± 2.91
Comp. **2** [44 μmol/L]	67.49 ± 4.12 ***	1.78 ± 1.84	9.95 ± 3.93 *	20.74 ± 6.80 *
Comp. **2** [88 μmol/L]	51.39 ± 4.15 ***	1.19 ± 0.05 *	10.04 ± 4.90 *	37.38 ± 8.36 ***
Comp. **3** [72.5 μmol/L]	69.50 ± 5.08 **	0.88 ± 0.15 **	9.27 ± 4.24 ^#^	23.68 ± 12.42 *
Comp. **3** [145 μmol/L]	21.15 ± 3.92 **	0.56 ± 0.14 *	7.16 ± 1.92	71.13 ± 2.14 **
Comp. **4** [678.5 μmol/L]	61.14 ± 0.74 **	1.41 ± 0.30	12.41 ± 2.03 *	25.03 ± 1.60 ***
Comp. **4** [1357 μmol/mL]	31.32 ± 4.67 **	2.44 ± 0.65 *	7.91 ± 1.32 *	58.32 ± 4.58 **
Comp. **8** [7.8 μmol/L]	70.75 ± 2.68 **	1.74 ± 0.29	7.34 ± 2.01 *	23.90 ± 3.64 **
Comp. **8** [15.6 μmol/L]	30.72 ± 1.10 **	1.07 ± 0.09 *	9.07 ± 0.26 *	59.14 ± 1.21 ***
Comp. **10** [24.2 μmol/L]	65.58 ± 3.41 **	1.64 ± 0.24	9.57 ± 1.67 *	23.21 ± 1.99 **
Comp. **10** [48.5 μmol/L]	47.48 ± 1.70 **	1.93 ± 0.22	8.93 ± 0.22	41.65 ± 1.05 **

MCF-7 cells were treated with compounds **2**, **3**, **4**, **8** and **10** at concentrations corresponding to ½ × IC_50_, and IC_50_ values for 24 h followed by staining with Annexin V FITC and propidium iodide (PI). (E − −)—living cells, (E − +)—necrotic cells; (E + −)—early-apoptotic cells; (E + +)—late-apoptotic cells. Results are presented as the mean ± SD, *n* = 3–6. Statistically significant differences between the samples and respective controls are depicted by asterisk (* *p* < 0.05; ** *p* < 0.01; *** *p* < 0.001); ^#^ *p* = 0.064.

**Table 5:** Annexin V-FITC/PI double staining analysis of apoptosis in MDA-MB-231 cells.

Compound [μmol/L]	Living Cells [E − −] [%]	Necrotic Cells [E − +] [%]	Early Apoptotic [E + −] [%]	Late Apoptotic [E + +] [%]
Control MDA-MB-231	86.20 ± 5.45	1.92 ± 0.60	6.12 ± 2.94	5.75 ± 2.37
Comp. **4** [654 μmol/L]	80.76 ± 2.22 *	1.61 ± 0.51	4.78 ± 0.62 *	12.84 ± 1.40 **
Comp. **4** [1308 μmol/L]	49.68 ± 1.41 ***	1.10 ± 0.11	3.90 ± 0.39 *	45.36 ± 1.64 ***
Comp. **8** [452 μmol/L]	80.75 ± 0.92	0.60 ± 0.36 **	1.61 ± 1.17 *	17.02 ± 2.39 *
Comp. **8** [904 μmol/L]	34.94 ± 8.78 **	4.04 ± 0.80	3.07 ± 1.06 **	57.94 ± 8.65 **
Comp. **10** [496 μmol/L]	69.21 ± 0.89 *	4.50 ± 0.89 *	3.94 ± 0.59 *	22.34 ± 1.48 *
Comp. **10** [992 μmol/mL]	50.44 ± 4.35 **	3.65 ± 0.39 *	5.01 ± 0.42 **	40.90 ± 4.04 **

MDA-MB-231 cells were treated with compounds **4**, **8** and **10** at concentrations corresponding to ½ × IC_50_, and IC_50_ values for 24 h followed by staining with Annexin V FITC and propidium iodide (PI). (E − −)—living cells, (E − +)—necrotic cells; (E + −)—early-apoptotic cells; (E + +)—late-apoptotic cells. Results are presented as the mean ± SD, *n* = 3. Statistically significant differences between the samples and respective controls are depicted by asterisk (* *p* < 0.05; ** *p* < 0.01; *** *p* < 0.001).

**Table 6:** Effects of selected biguanides on cell cycle progression in MCF-7 and MDA-MB-231 cells.

Compound [µmol/L]	Sub G0/G1 [%]	G0/G1 [%]	S [%]	G2/M [%]
Control MCF-7	1.24 ± 0.70	52.30 ± 1.84	26.01 ± 0.70	20.45 ± 1.30
Comp. **2** [88 µmol/L]	1.55 ± 0.18	69.69 ± 0.94 ***	15.42 ± 0.71 ***	13.28 ± 0.38 ***
Comp. **3** [145 µmol/L]	1.47 ± 0.07	70.34 ± 0.71 ***	14.89 ± 0.38 ***	13.27 ± 0.58 ***
Comp. **4** [1357 µmol/L]	1.96 ± 0.60	70.63 ± 1.47 ***	12.82 ± 0.50 ***	14.54 ± 0.31 ***
Comp. **8** [15.6 µmol/L]	2.75 ± 0.51 ***	72.79 ± 0.56 ***	12.83 ± 1.19 ***	11.61 ± 0.74 ***
Comp. **10** [48.5 µmol/L]	1.71 ± 0.19	68.23 ± 0.60 ***	15.52 ± 0.22 ***	14.54 ± 0.43 ***
Control MDA-MB-231	1.40 ± 0.14	57.09 ± 0.91	23.27 ± 0.65	18.20 ± 0.99
Comp. **4** [1308 µmol/L]	1.77 ± 0.19	65.77 ± 0.93	20.95 ± 0.71	11.54 ± 1.11 ***
Comp. **8** [904 µmol/L]	1.83 ± 0.21 *	70.32 ± 1.95 **	16.61 ± 1.36 **	11.20 ± 0.96 ***
Comp. **10** [992 µmol/L]	1.73 ± 0.19	60.65 ± 7.04	20.65 ± 1.05 *	13.07 ± 0.30 ***

MCF-7 cells were treated with tested compounds **2**, **3**, **4**, **8** and **10** at concentrations corresponding to IC_50_ values for 24 h followed by staining with propidium iodide (PI). MDA-MB-231 cells were treated with compounds **4**, **8** and **10** (concentrations equaling IC_50_ value). DNA content was determined using a flow cytometry analysis of PI stained cells. Results are presented as the mean ± SD, *n* = 3. Statistically significant differences between the samples and respective controls are depicted by asterisks (* *p* < 0.05; ** *p* < 0.01; *** *p* < 0.001).

## Data Availability

The datasets generated during the current study are available from the corresponding author on reasonable request.

## Supplementary Materials

The following are available online at https://www.mdpi.com/article/10.3390/ijms22115642/s1.
