# Supplementary material for: Incorporation of Sulfonamide Moiety into Biguanide Scaffold Results in Apoptosis Induction and Cell Cycle Arrest in MCF-7 Breast Cancer Cells
Source: Int J Mol Sci. 2021 May 26;22(11):5642. doi: 10.3390/ijms22115642 (PMC8198066; doi:10.3390/ijms22115642)

SUPPORTING INFORMATION

# Incorporation of sulfonamide moiety into biguanide scaffold results in apoptosis induction and cell cycle arrest in MCF-7 breast cancer cells.

**Magdalena Markowicz-Piasecka[1*], Karol Sadowski [2], Johanna Huttunen [3], Joanna Sikora [1] and Kristiina M. Huttunen [3]**

[1] Laboratory of Bioanalysis, Department of Pharmaceutical Chemistry, Drug Analysis and Radiopharmacy, Medical University of Lodz, ul. Muszyńskiego1, 90-151 Lodz, Poland; e-mail: magdalena.markowicz@umed.lodz.pl; joanna.sikora@umed.lodz.pl
[2] Students Research Group, Department of Pharmaceutical Chemistry, Drug Analysis and Radiopharmacy, Medical University of Lodz, ul. Muszyńskiego1, 90-151 Lodz, Poland; e-mail: karol.sadowski1@stud.umed.lodz.pl
[3] School of Pharmacy, Faculty of Health Sciences, University of Eastern Finland, Yliopistonranta 1C, POB 1627, 70211 Kuopio, Finland; e-mail: kristiina.huttunen@uef.fi

**Table S1.** Eadie-Hofstee analysis of the uptake of sulfonamide derivatives of metformin (**1 – 10**) in MCF-7 and MDA-MB-231 cells ($K_m$ and $V_{max}$ values).

| | | Kinetic parameters of prodrugs uptake | | | |
|---|---|---|---|---|---|
| | | **MCF-7** | | **MDA-231 cells** | |
| **Compound** | **Transp.** | $\mathbf{K_m}$ [μmol/L] | $\mathbf{V_{max}}$ [nmol/min/mg] | $\mathbf{K_m}$ [μmol/L] | $\mathbf{V_{max}}$ [nmol/min/mg] |
| **1** | I | 890.4 ± 216.1 | 0.653 ± 0.124 | 22.66 ± 3.0 | 0.476 ± 0.023 |
| | $V_{max}/K_m$ | 0.000733 | | 0.02101 | |
| | II | 1054 ± 556.2 | 1.225 ± 0.358 | 298.4 ± 134.0 | 0.714 ± 0.184 |
| | $V_{max}/K_m$ | 0.001162 | | 0.002393 | |
| **2** | I | 257.9 ± 43.28 | 1.147 ± 0.084 | 2115 ± 470.7 | 0.958 ± 0.149 |
| | $V_{max}/K_m$ | 0.004447 | | 0.000453 | |
| | II | 1907 ± 490.0 | 4.640 ± 0.689 | 590.6 ± 164.1 | 0.707 ± 0.079 |
| | $V_{max}/K_m$ | 0.002433 | | 0.001197 | |
| **3** | I | 532.7 ± 90.2 | 1.621 ± 0.151 | NE | NE |
| | $V_{max}/K_m$ | 0.003043 | | NE | |
| | II | 367.4 ± 75.1 | 1.856 ± 0.136 | NE | NE |
| | $V_{max}/K_m$ | 0.005052 | | NE | |
| **4** | I | 1518 ± 180.4 | 1.201 ± 0.121 | 144.6 ± 32.81 | 0.249 ± 0.022 |
| | $V_{max}/K_m$ | 0.000791 | | 0.001722 | |
| | II | 3389 ± 605.9 | 2.850 ± 0.438 | 2930 ± 652.4 | 1.504 ± 0.278 |
| | $V_{max}/K_m$ | 0.000841 | | 0.000513 | |
| **5** | I | 413.5 ± 167.2 | 0.491 ± 0.182 | 31.18 ± 9.06 | 0.274 ± 0.023 |
| | $V_{max}/K_m$ | 0.001187 | | 0.008788 | |
| | II | 760.3 ± 86.20 | 1.160 ± 0.080 | 693.8 ± 311.8 | 1.021 ± 0.211 |
| | $V_{max}/K_m$ | 0.001526 | | 0.001472 | |
| **6** | I | 1260 ± 178.3 | 2.713 ± 0.235 | NE | NE |
| | $V_{max}/K_m$ | 0.002153 | | NE | |
| | II | 1077 ± 145.1 | 2.705 ± 0.234 | NE | NE |
| | $V_{max}/K_m$ | 0.002512 | | NE | |
| **7** | I | 588.5 ± 79.7 | 2.223 ± 0.154 | 2689 ± 1945 | 0.569 ± 0.290 |
| | $V_{max}/K_m$ | 0.003777 | | 0.000212 | |
| | II | 962.7 ± 60.78 | 3.980 ± 0.140 | 349.5 ± 70.54 | 0.313 ± 0.029 |
| | $V_{max}/K_m$ | 0.004134 | | 0.000895 | |
| **8** | I | 358.1 ± 256.7 | 0.629 ± 0.223 | 1755 ± 418.1 | 38.20 ± 6.451 |
| | $V_{max}/K_m$ | 0.001756 | | 0.021766 | |
| | II | 896.7 ± 213.3 | 2.802 ± 0.276 | 546.5 ± 358.3 | 27.91 ± 6.767 |
| | $V_{max}/K_m$ | 0.003124 | | 0.051070 | |
| **9** | I | 259.7 ± 63.31 | 0.793 ± 0.099 | 630.5 ± 466.6 | 0.885 ± 0.412 |
| | $V_{max}/K_m$ | 0.003053 | | 0.001404 | |
| | II | 465.8 ± 188.3 | 1.296 ± 0.169 | 137.2 ± 240.2 | 1.028 ± 0.463 |
| | $V_{max}/K_m$ | 0.002782 | | 0.007493 | |
| **10** | I | NE | NE | NE | NE |

| | | | | | |
|---|---|---|---|---|---|
| | $V_{max}/K_m$ | NE | | NE | |
| | II | NE | NE | NE | NE |
| | $V_{max}/K_m$ | NE | | NE | |

NE- not established.

**Figure S1.** The uptake of compounds **3** - **10** into MCF-7 cells at the concentrations of 10 – 2000 µmol/L and Eadie–Hofstee plots for OCTs mediated transport.

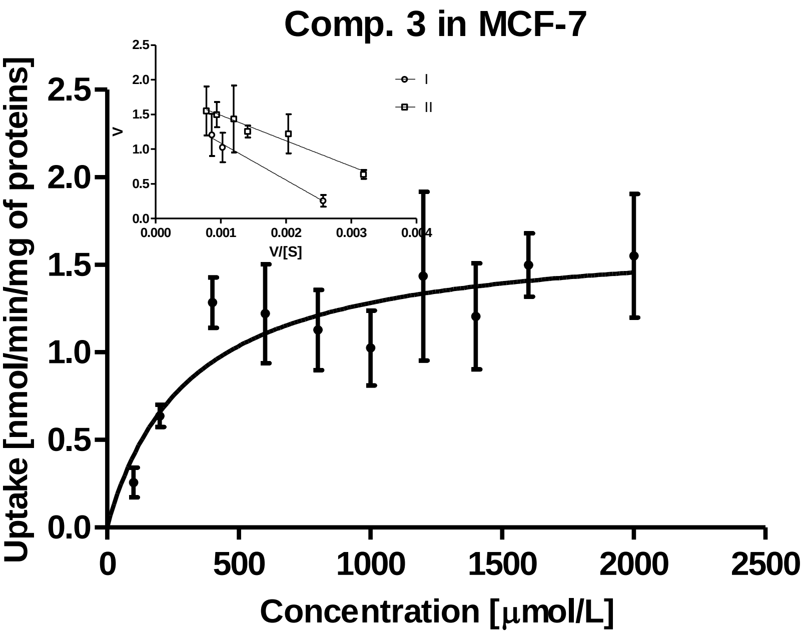

Comp. 4 in MCF-7

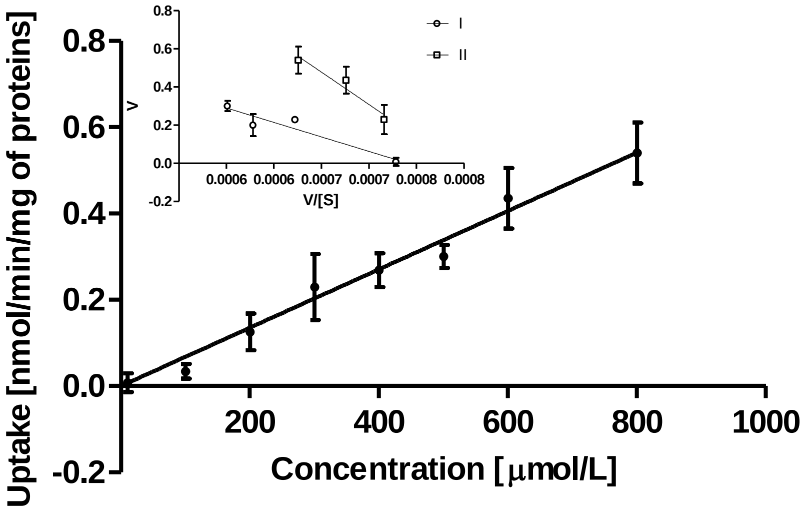


Comp. 5 in MCF-7 cells

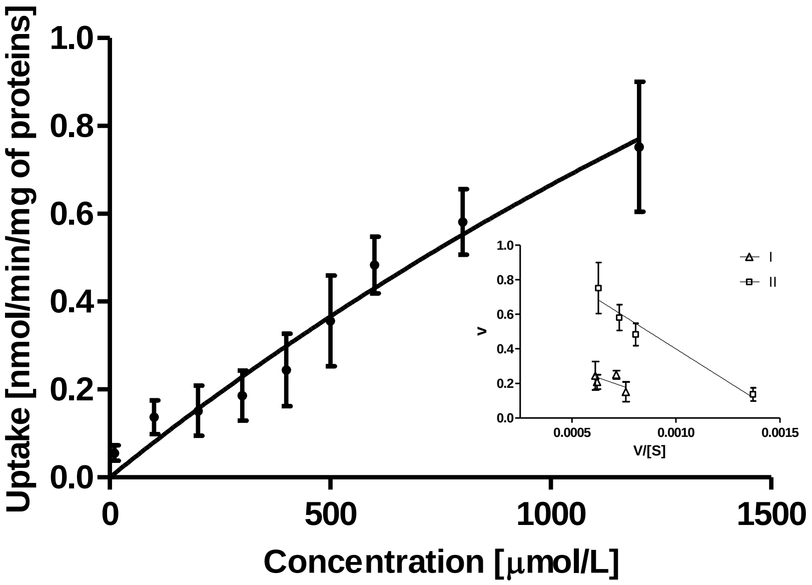

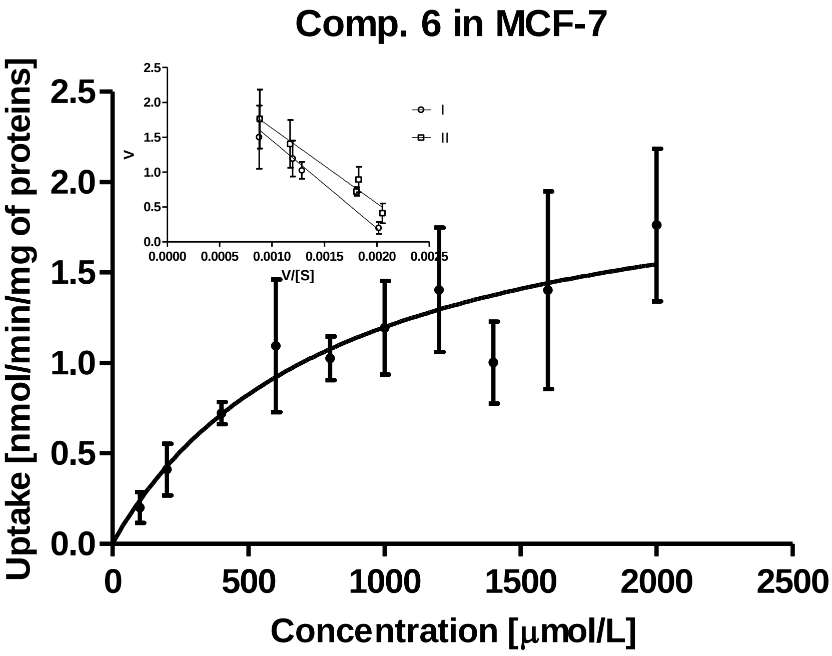
Comp. 6 in MCF-7
Uptake [nmol/min/mg of proteins]
2.5
2.0
1.5
1.0
0.5
0.0
0
500
1000
1500
2000
2500
Concentration [µmol/L]
V
V/[S]
I
II
0.0000
0.0005
0.0010
0.0015
0.0020
0.0025

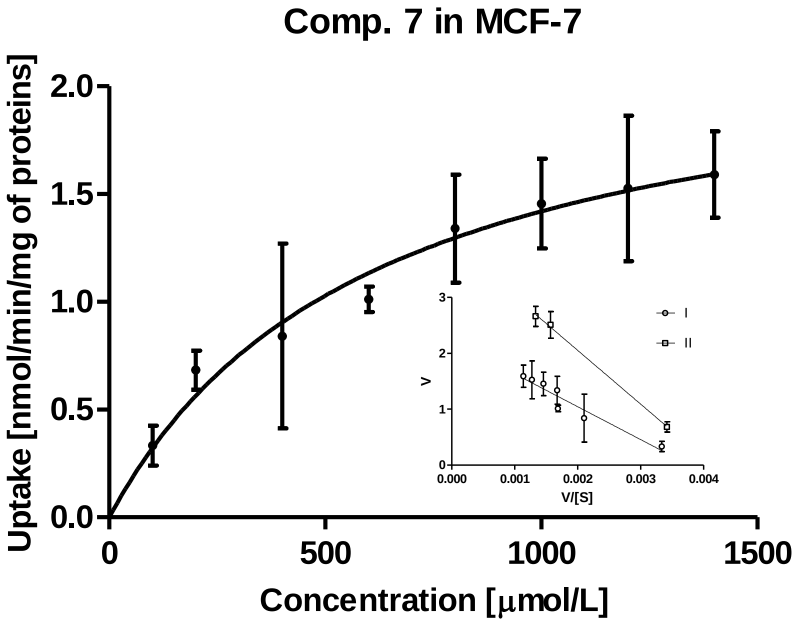
Comp. 7 in MCF-7
Uptake [nmol/min/mg of proteins]
2.0
1.5
1.0
0.5
0.0
0
500
1000
1500
Concentration [µmol/L]
V
V/[S]
I
II
0.000
0.001
0.002
0.003
0.004

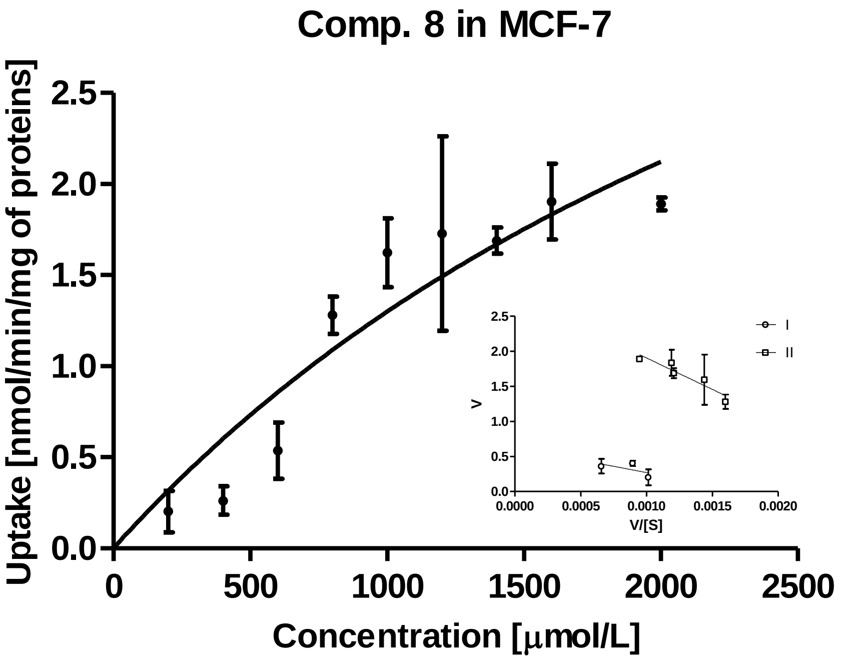
Comp. 8 in MCF-7
Uptake [nmol/min/mg of proteins]
Concentration [μmol/L]
0.0
0.5
1.0
1.5
2.0
2.5
0
500
1000
1500
2000
2500
I
II
V
V/[S]
0.0000
0.0005
0.0010
0.0015
0.0020

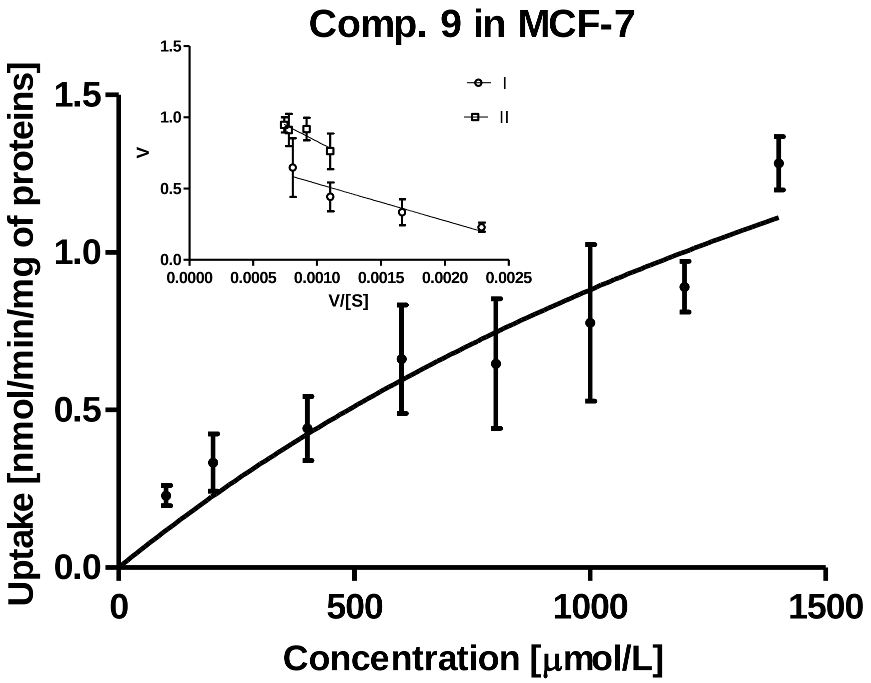
Comp. 9 in MCF-7
Uptake [nmol/min/mg of proteins]
Concentration [μmol/L]
0.0
0.5
1.0
1.5
0
500
1000
1500
I
II
V
V/[S]
0.0000
0.0005
0.0010
0.0015
0.0020
0.0025

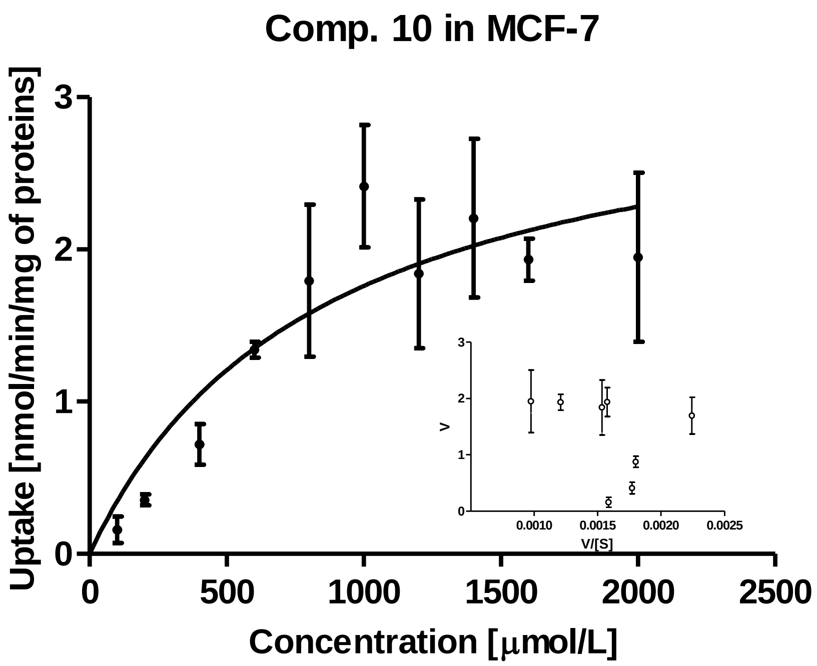
Comp. 10 in MCF-7
Uptake [nmol/min/mg of proteins]
Concentration [μmol/L]
0
1
2
3
0
500
1000
1500
2000
2500
V
V/[S]
0.0010
0.0015
0.0020
0.0025

**Figure S2.** The uptake mechanism of compounds **3 – 10** (400 and 800 µmol/L) into MCF-7 cells and MDA-MB-231 cells. The uptake was determined in the presence of OCT and MATE inhibitors, disopyramide, lopinavir, methenamine, cimetidine (400 and 800 µmol/L) for 10 minutes at 37 °C. The significant differences between pure compounds **3 – 10** and their mixtures with inhibitors are denoted with asterisk. * $p < 0.05$, ** $p < 0.01$, *** $p < 0.001$.

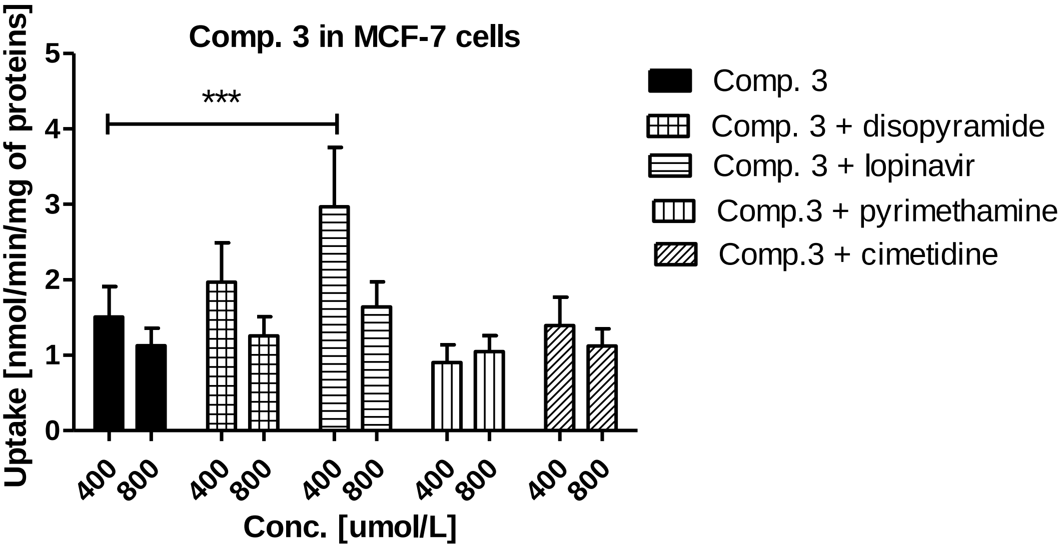


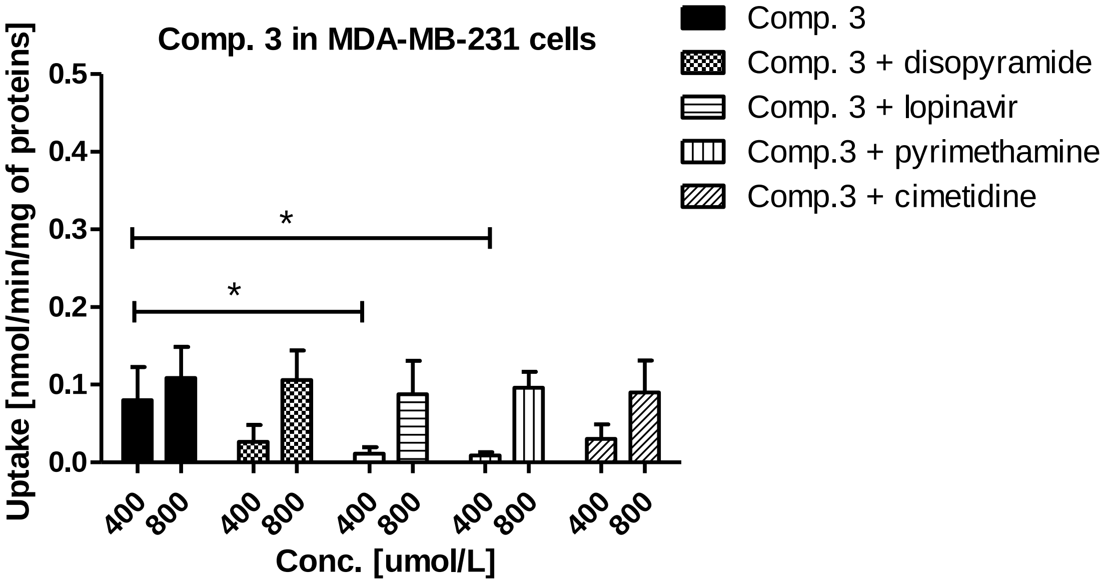

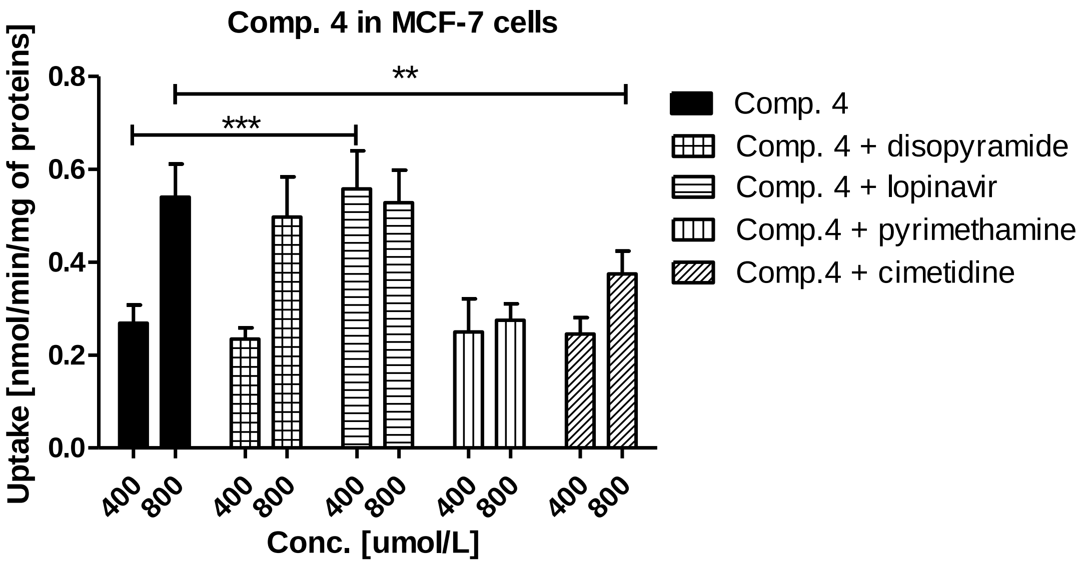

Comp. 4 in MCF-7 cells
Uptake [nmol/min/mg of proteins]
0.8
0.6
0.4
0.2
0.0
***
**
400
800
400
800
400
800
400
800
400
800
Conc. [umol/L]
Comp. 4
Comp. 4 + disopyramide
Comp. 4 + lopinavir
Comp.4 + pyrimethamine
Comp.4 + cimetidine


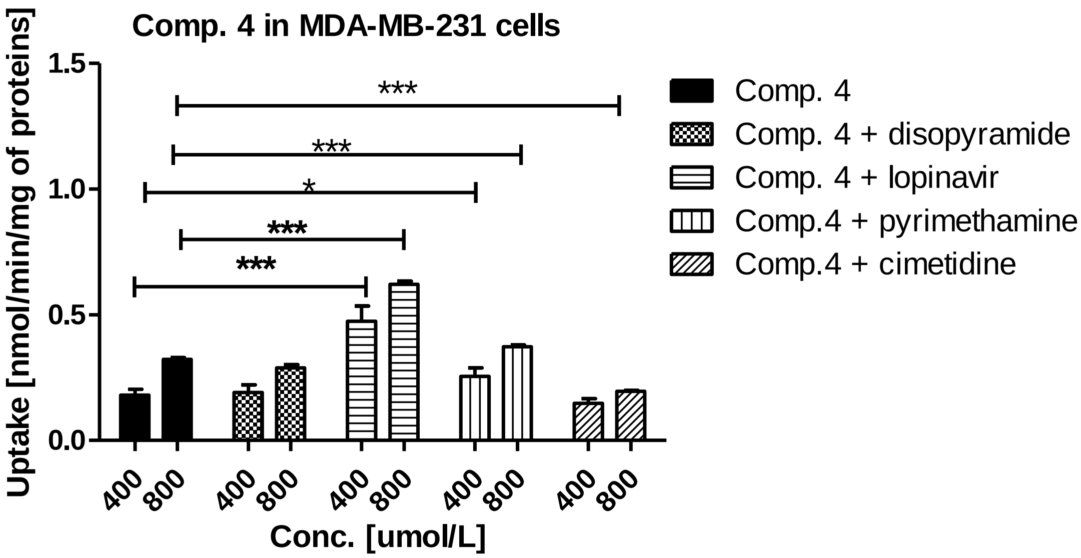

Comp. 4 in MDA-MB-231 cells
Uptake [nmol/min/mg of proteins]
1.5
1.0
0.5
0.0
***
***
*
***
***
400
800
400
800
400
800
400
800
400
800
Conc. [umol/L]
Comp. 4
Comp. 4 + disopyramide
Comp. 4 + lopinavir
Comp.4 + pyrimethamine
Comp.4 + cimetidine

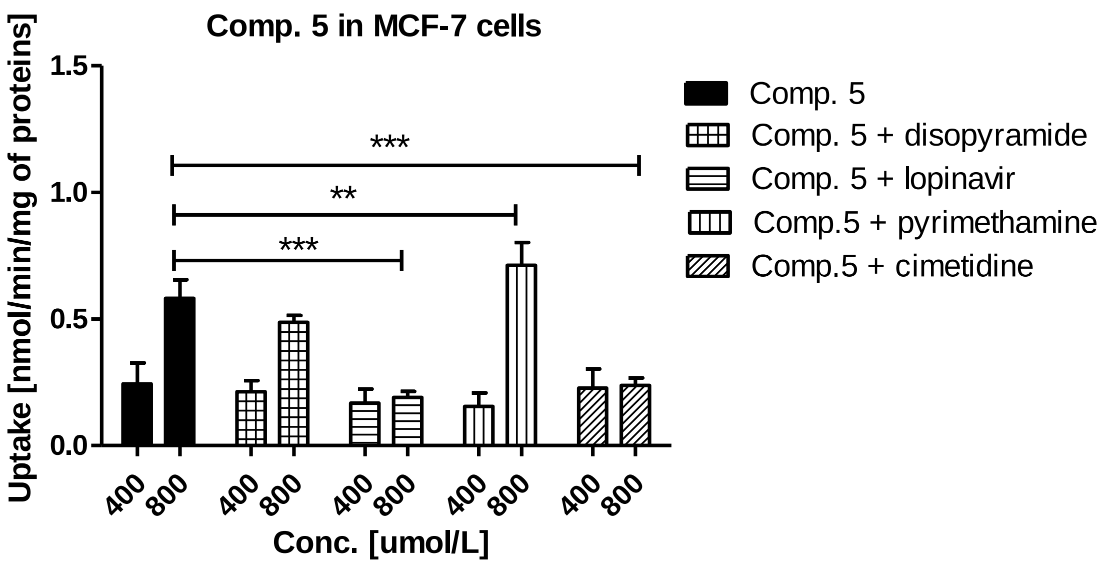
Comp. 5 in MCF-7 cells
Uptake [nmol/min/mg of proteins]
1.5
1.0
0.5
0.0
***
**
***
400
800
Conc. [umol/L]
Comp. 5
Comp. 5 + disopyramide
Comp. 5 + lopinavir
Comp.5 + pyrimethamine
Comp.5 + cimetidine

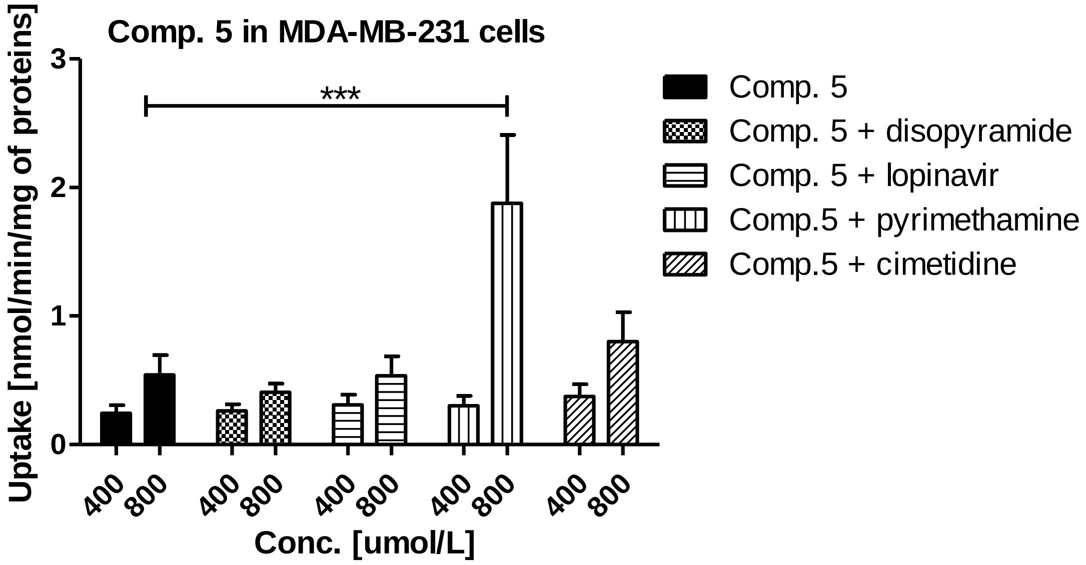
Comp. 5 in MDA-MB-231 cells
Uptake [nmol/min/mg of proteins]
3
2
1
0
***
400
800
Conc. [umol/L]
Comp. 5
Comp. 5 + disopyramide
Comp. 5 + lopinavir
Comp.5 + pyrimethamine
Comp.5 + cimetidine

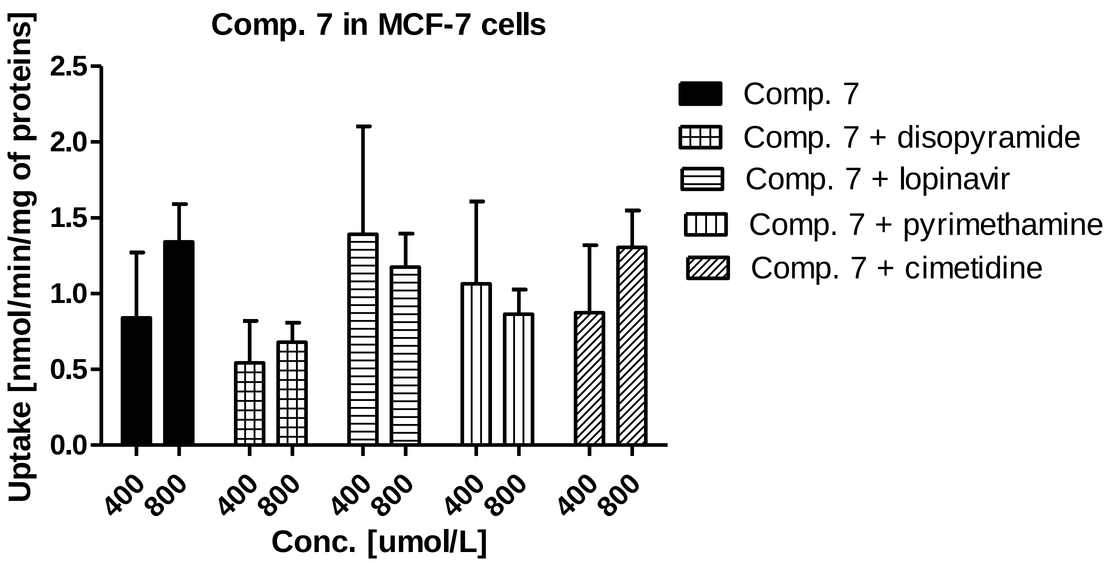
Comp. 7 in MCF-7 cells
Uptake [nmol/min/mg of proteins]
2.5
2.0
1.5
1.0
0.5
0.0
400
800
Conc. [umol/L]
Comp. 7
Comp. 7 + disopyramide
Comp. 7 + lopinavir
Comp. 7 + pyrimethamine
Comp. 7 + cimetidine

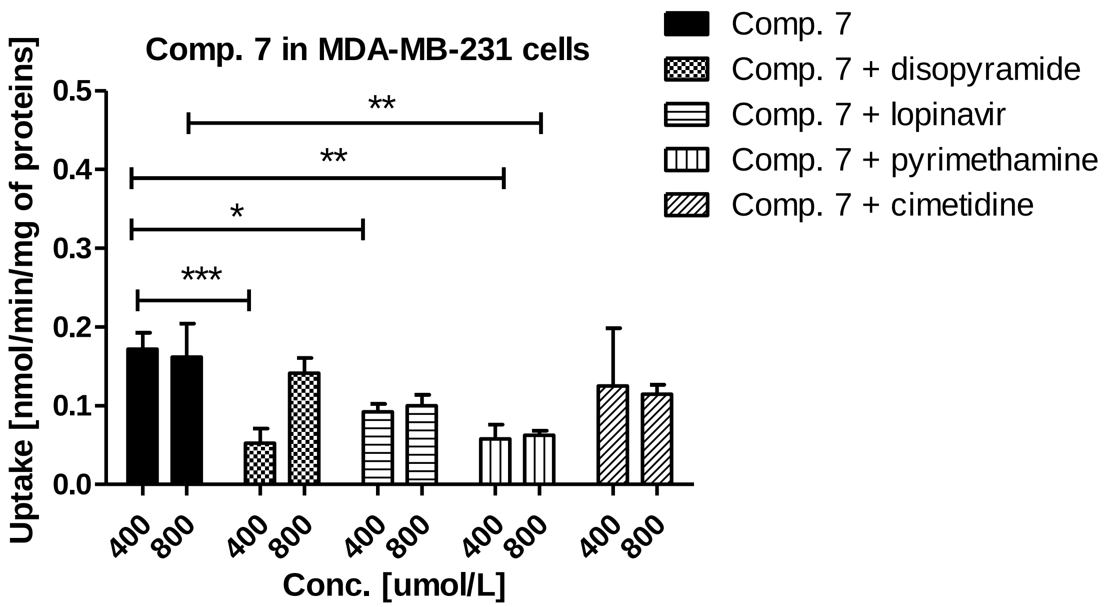
Comp. 7 in MDA-MB-231 cells
Uptake [nmol/min/mg of proteins]
0.5
0.4
0.3
0.2
0.1
0.0
**
**
*
***
400
800
Conc. [umol/L]
Comp. 7
Comp. 7 + disopyramide
Comp. 7 + lopinavir
Comp. 7 + pyrimethamine
Comp. 7 + cimetidine

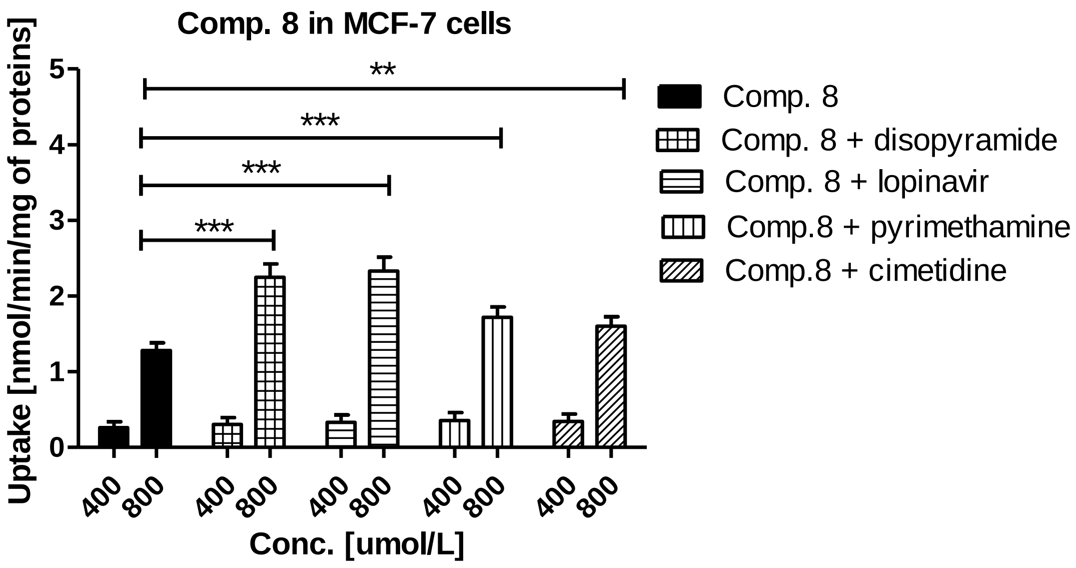
Comp. 8 in MCF-7 cells
Uptake [nmol/min/mg of proteins]
0
1
2
3
4
5
**
***
***
***
400
800
400
800
400
800
400
800
400
800
Conc. [umol/L]
Comp. 8
Comp. 8 + disopyramide
Comp. 8 + lopinavir
Comp.8 + pyrimethamine
Comp.8 + cimetidine

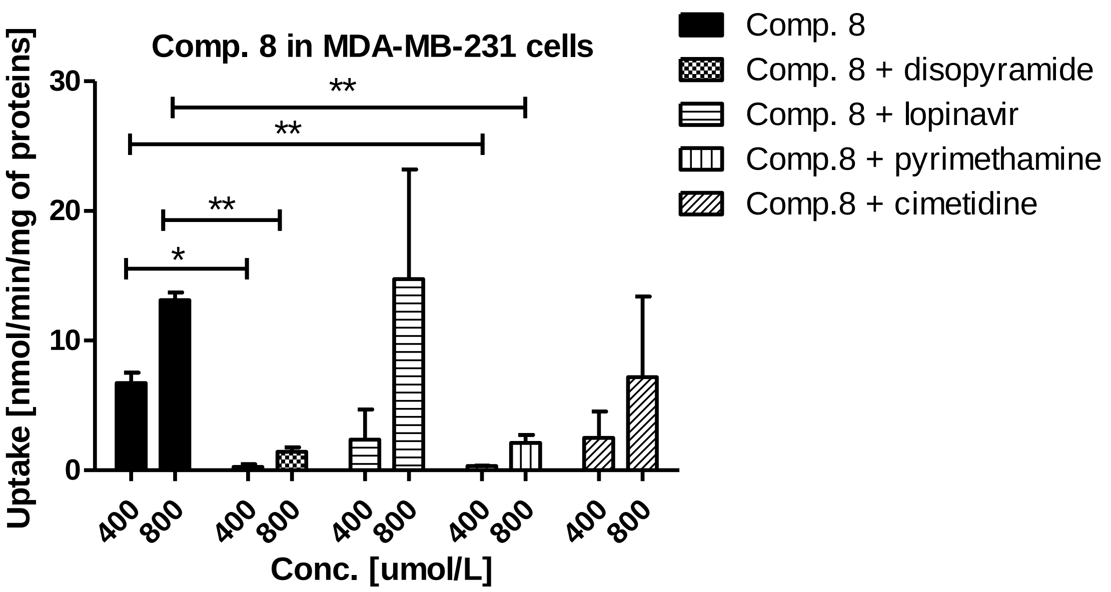
Comp. 8 in MDA-MB-231 cells
Uptake [nmol/min/mg of proteins]
0
10
20
30
**
**
**
*
400
800
400
800
400
800
400
800
400
800
Conc. [umol/L]
Comp. 8
Comp. 8 + disopyramide
Comp. 8 + lopinavir
Comp.8 + pyrimethamine
Comp.8 + cimetidine

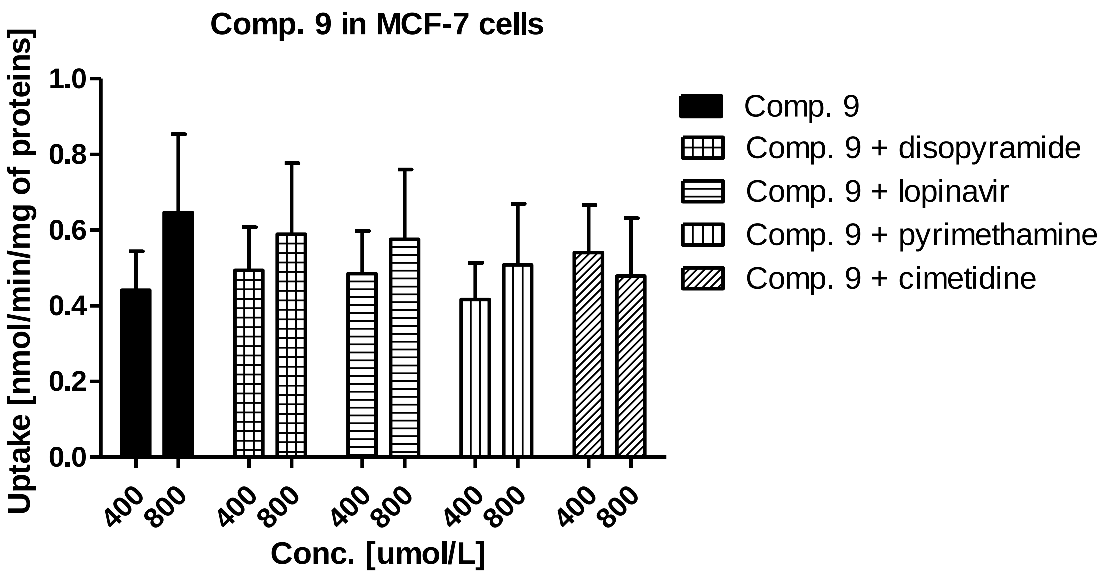
Comp. 9 in MCF-7 cells
Uptake [nmol/min/mg of proteins]
1.0
0.8
0.6
0.4
0.2
0.0
400 800 400 800 400 800 400 800 400 800
Conc. [umol/L]
Comp. 9
Comp. 9 + disopyramide
Comp. 9 + lopinavir
Comp. 9 + pyrimethamine
Comp. 9 + cimetidine

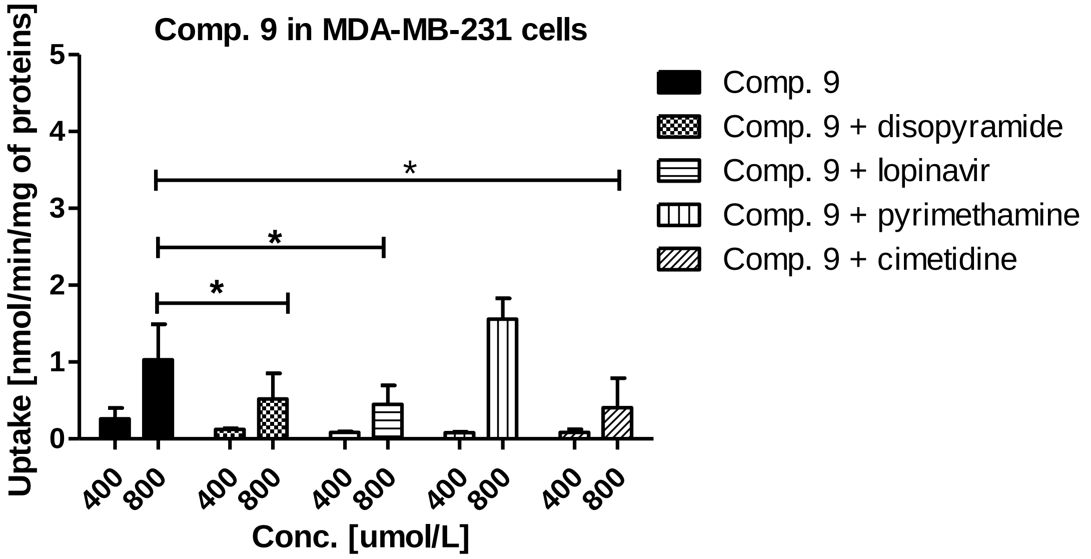
Comp. 9 in MDA-MB-231 cells
Uptake [nmol/min/mg of proteins]
5
4
3
2
1
0
*
*
*
400 800 400 800 400 800 400 800 400 800
Conc. [umol/L]
Comp. 9
Comp. 9 + disopyramide
Comp. 9 + lopinavir
Comp. 9 + pyrimethamine
Comp. 9 + cimetidine

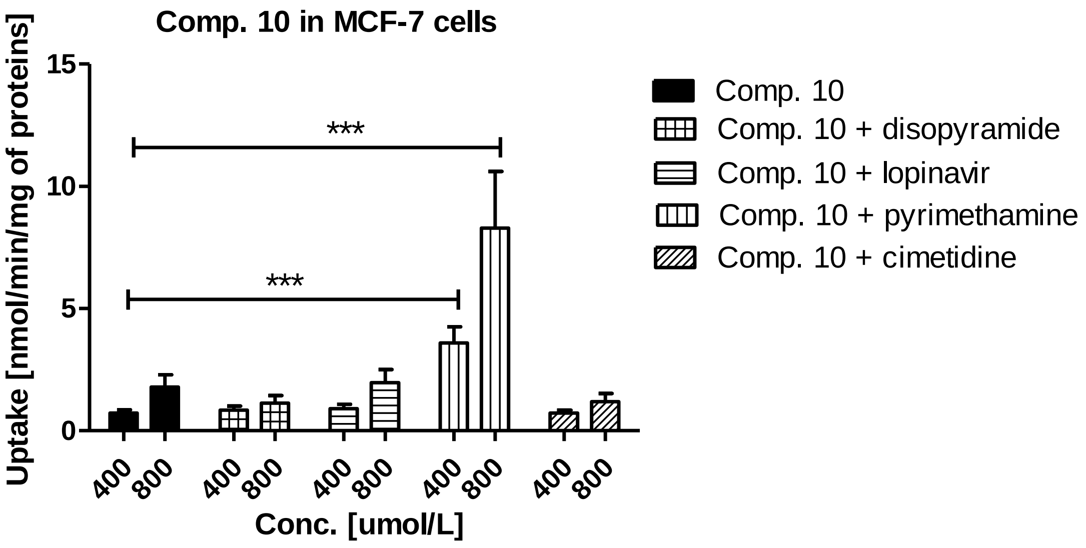

Comp. 10 in MCF-7 cells
Uptake [nmol/min/mg of proteins]
15
10
5
0
***
***
400
800
Conc. [umol/L]
Comp. 10
Comp. 10 + disopyramide
Comp. 10 + lopinavir
Comp. 10 + pyrimethamine
Comp. 10 + cimetidine


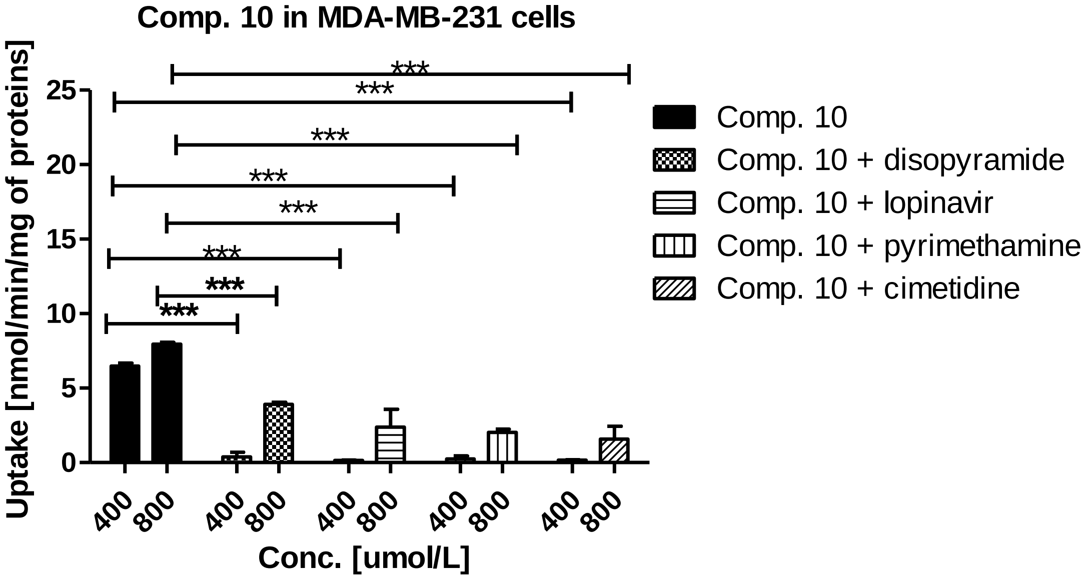

Comp. 10 in MDA-MB-231 cells
Uptake [nmol/min/mg of proteins]
25
20
15
10
5
0
***
400
800
Conc. [umol/L]
Comp. 10
Comp. 10 + disopyramide
Comp. 10 + lopinavir
Comp. 10 + pyrimethamine
Comp. 10 + cimetidine

**Figure S3.** The uptake of compounds **3** - **10** into MDA-MB-231 cells at the concentrations of 10 – 2000 μmol/L and Eadie–Hofstee plots for OCTs mediated transport.

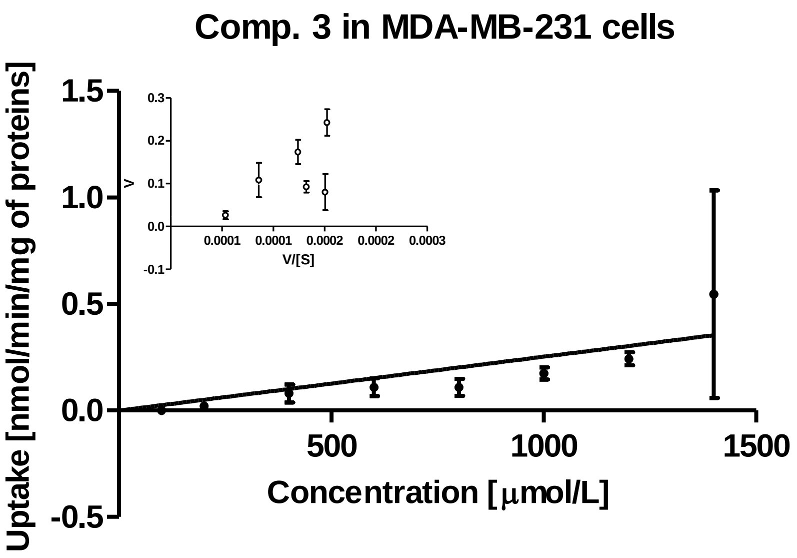


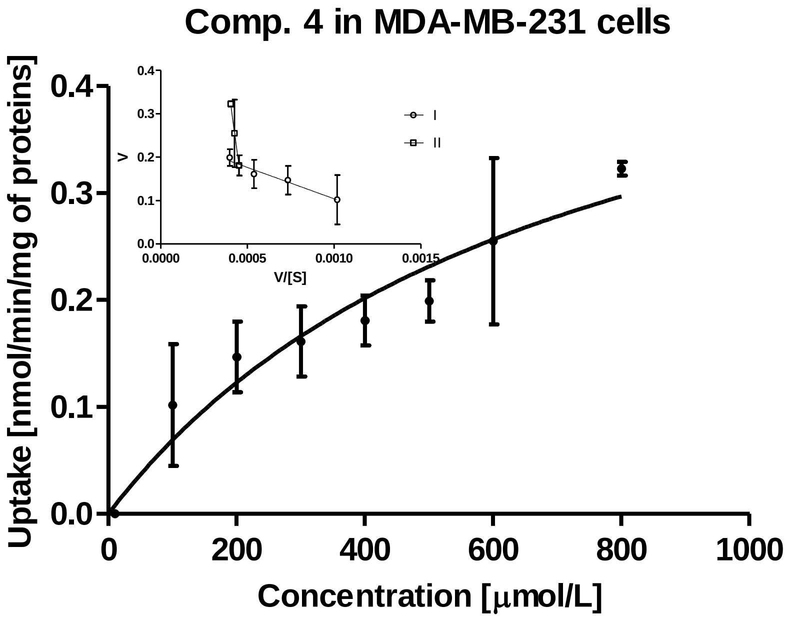

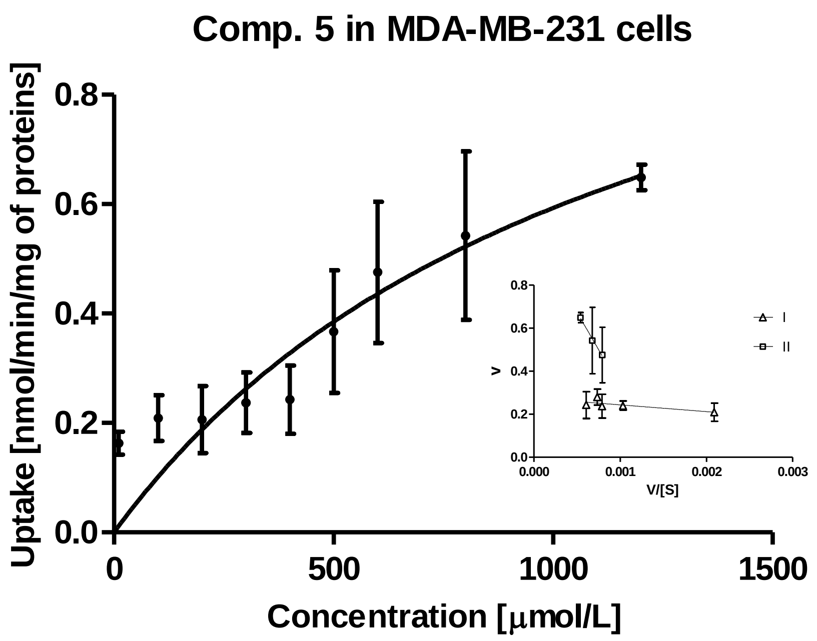
Comp. 5 in MDA-MB-231 cells
Uptake [nmol/min/mg of proteins]
0.8
0.6
0.4
0.2
0.0
0
500
1000
1500
Concentration [μmol/L]
V
V/[S]
I
II
0.000
0.001
0.002
0.003

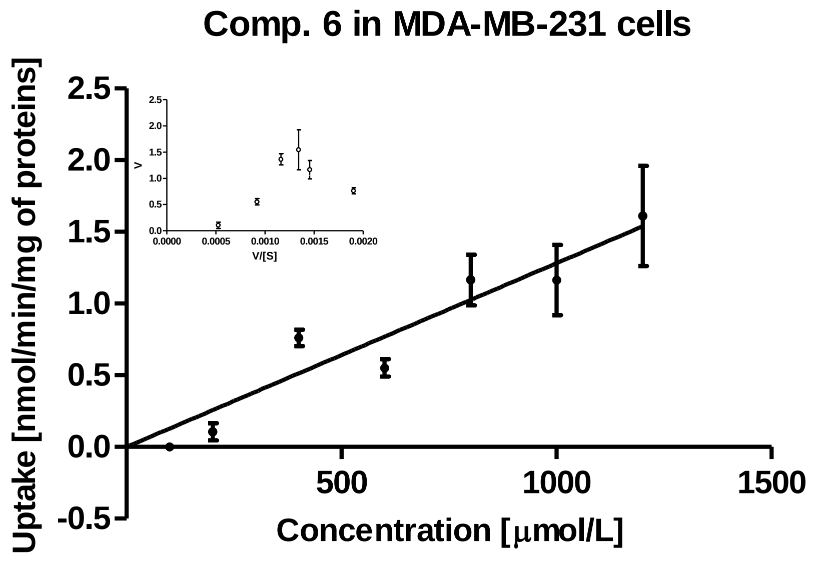
Comp. 6 in MDA-MB-231 cells
Uptake [nmol/min/mg of proteins]
2.5
2.0
1.5
1.0
0.5
0.0
-0.5
500
1000
1500
Concentration [μmol/L]
V
V/[S]
0.0000
0.0005
0.0010
0.0015
0.0020

## Comp. 7 in MDA-MB-231 cells

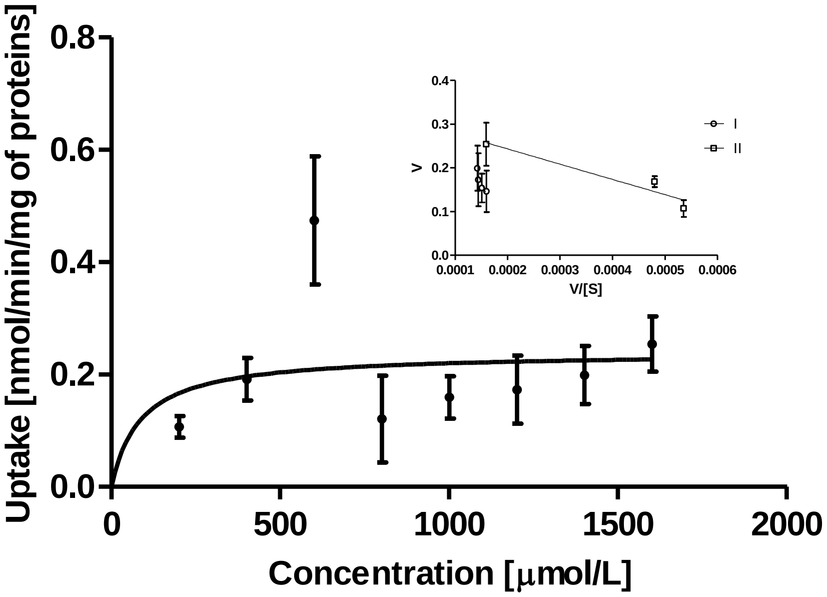


## Comp. 8 in MDA-MB-231 cells

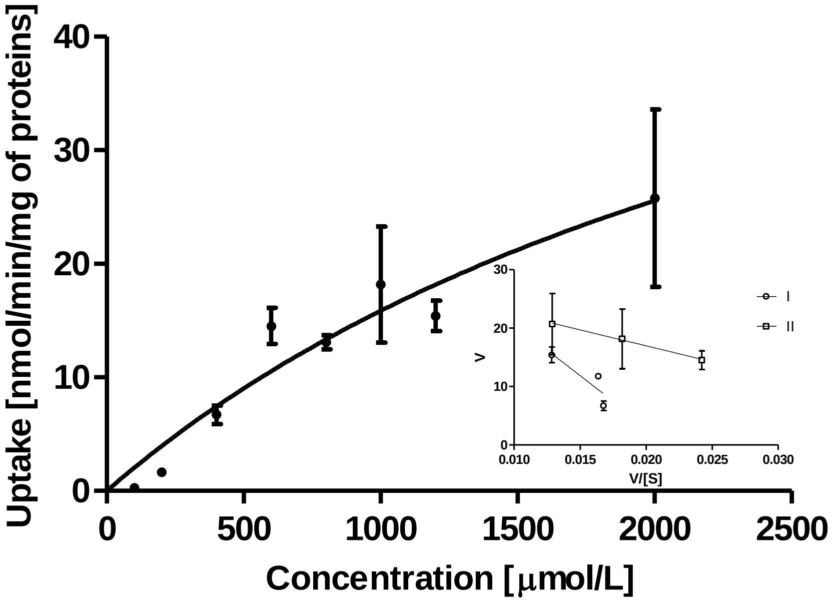

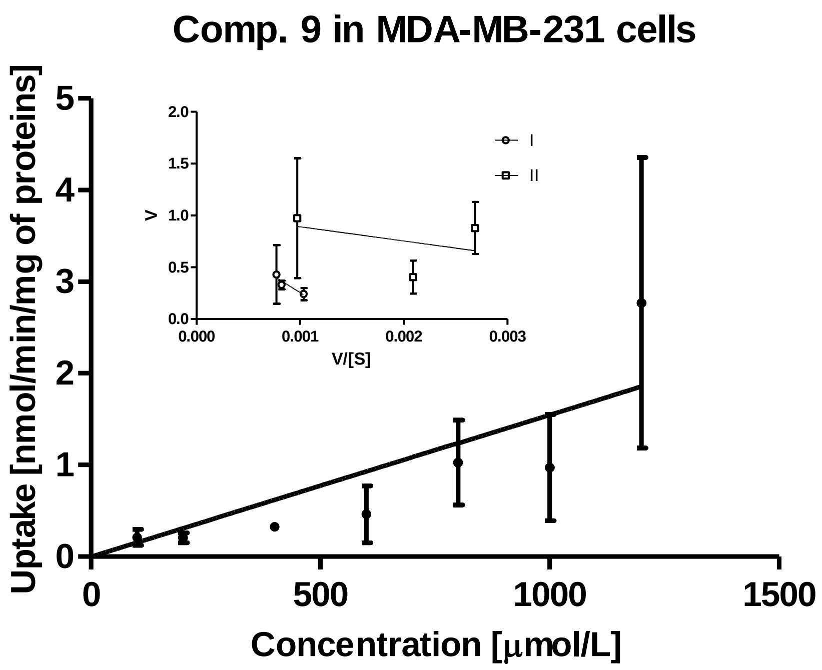

Comp. 9 in MDA-MB-231 cells
Uptake [nmol/min/mg of proteins]
Concentration [µmol/L]
0
500
1000
1500
0
1
2
3
4
5
V
V/[S]
I
II
0.0
0.5
1.0
1.5
2.0
0.000
0.001
0.002
0.003


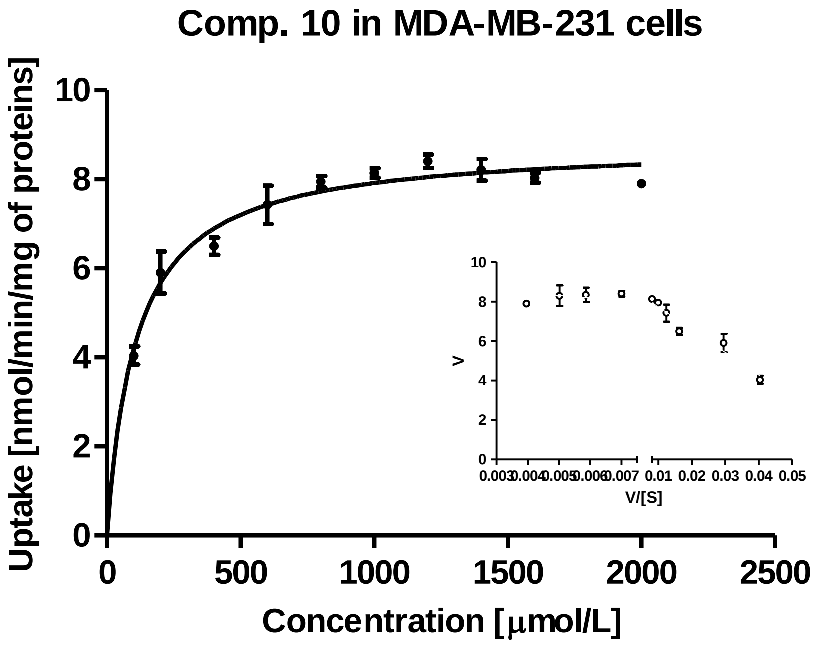

Comp. 10 in MDA-MB-231 cells
Uptake [nmol/min/mg of proteins]
Concentration [µmol/L]
0
500
1000
1500
2000
2500
0
2
4
6
8
10
V
V/[S]
0.003
0.004
0.005
0.006
0.007
0.01
0.02
0.03
0.04
0.05

**Table S2.** The summary of interactions of metformin derivatives 1 – 10 with OCT, PMAT and MATE1 transporters in MCF-7 and MDA-MB-231 cells.

| COMPOUND | MCF-7 | MDA-MB-231 |
|---|---|---|
| **1** | OCT3 (non-sign.) | OCT3, PMAT |
| **2** | PMAT, MATE1 | OCT1, PMAT |
| **3** | PMAT | PMAT, MATE1 |
| **4** | OCT3, PMAT | OCT3, PMAT, MATE1 |
| **5** | OCT3, PMAT, MATE1 | MATE1 |
| **6** | OCT1, OCT3, PMAT, MATE1 | OCT1, MATE1 |
| **7** | OCT1 (non-sign.) | OCT1, PMAT, MATE1 |
| **8** | OCT1, OCT3, PMAT, MATE1 | OCT1, MATE1 |
| **9** | OCT3 (non-sign.) | OCT1, OCT3, PMAT |
| **10** | MATE1 | OCT1, OCT3, PMAT, MATE1 |

**Table S3.** Effects of selected biguanides on the production of intracellular reactive oxygen species in MCF-7 and MDA-MB-231 cells.

| Compound [μmol/L] | Median fluorescence [RFU] |
|---|---|
| **Control MCF-7** | **204 422.3 ± 17 741.1** |
| AAPH [100 μmol/L] | 328 183.3 ± 95 164.4* |
| Metformin [500 μmol/L] | 226 427.2 ± 60 332.4 |
| Comp. **2** [44 μmol/L] | 40 907.4 ± 10 098.9** |
| Comp. **3** [72.5 μmol/L] | 391 177.4 ± 42 746.8* |
| Comp. **4** [678.5 μmol/L] | 179 072.7 ± 22 957.6 |
| Comp. **8** [7.8 μmol/L] | 418 376.2 ± 272 653.5 |
| Comp. **10** [24.2 μmol/L] | 377 063.6 ± 18 351.2** |
| **Control MDA-MB-231** | **184 715.4 ± 23 171.9** |
| AAPH [100 μmol/L] | 344 061.3 ± 65 766.2* |
| Metformin [500 μmol/L] | 305 732.8 ± 36 879.5* |
| Comp. 4 [678.5 μmol/L] | 198 801.63 ± 25 103.0 |
| Comp. 8 [452 μmol/L] | 252 568.2 ± 22 955.4 |
| Comp. 10 [496 μmol/L] | 171 794.2 ± 23 831.0 |

The experiments were performed using 2′,7′-dichlorodihydrofluorescein diacetate ($H_2$DCFDA), a fluorescent indicator for ROS in cells. The fluorescence of stained cells was measured by flow cytometry. 2,2'-Azobis(2-amidinopropane) dihydrochloride (AAPH) was used as positive control. Results are presented as median intensity of fluorescence of 10000 events (n = 3). Statistically significant differences between the samples and respective controls are depicted by asterisks ($p < 0.05$; ** $p < 0.01$).

**Table S4.** The effects of metformin and its sulfonamide derivatives on MCF-7 cell migration.

| Compound | Conc. [μmol/L] | $T_0$ [μm] | $T_4$ [μm] | $T_{24}$ [μm] | $T_{48}$ [μm] |
|---|---|---|---|---|---|
| **Control** | - | 77.05 ± 13.80 | 70.79 ± 10.80 | 56.84 ± 5.13 | 38.70 ± 8.11 |
| **Metformin** | 500 | 76.90 ± 4.48 | 78.67 ± 3.97 | 72.73 ± 3.89* | 60.97 ± 3.07*** |
| | 1000 | 77.77 ± 5.53 | 76.56 ± 3.56 | 73.11 ± 2.28* | 63.84 ± 7.12*** |
| **Comp. 2** | 22 | 83.74 ± 15.63 | 82.52 ± 10.61* | 83.29 ± 10.98*** | 76.68 ± 15.27*** |
| | 44 | 83.84 ± 11.67 | 82.46 ± 10.98* | 81.88 ± 10.47*** | 73.80 ± 12.07*** |
| **Comp. 3** | 36.2 | 83.44 ± 9.38 | 80.47 ± 7.09 | 71.36 ± 9.06** | 64.78 ± 15.91*** |
| | 72.5 | 72.65 ± 4.38 | 71.03 ± 5.54 | 68.74 ± 9.65* | 60.94 ± 16.82*** |
| **Comp. 4** | 339.25 | 83.99 ± 12.23 | 82.77 ± 9.49* | 66.12 ± 8.57 | 73.00 ± 12.01*** |
| | 678.5 | 76.76 ± 14.23 | 75.99 ± 13.63 | 76.62 ± 12.27*** | 71.00 ± 14.41*** |
| **Comp. 8** | 3.9 | 79.24 ± 13.03 | 78.09 ± 12.85 | 77.35 ± 15.31*** | 70.08 ± 15.92*** |
| | 7.8 | 80.79 ± 12.02 | 78.52 ± 11.69 | 70.98 ± 16.39** | 65.23 ± 16.19*** |
| **Comp. 10** | 12.1 | 86.52 ± 9.93 | 80.54 ± 10.81 | 73.10 ± 17.41** | 69.66 ± 16.38*** |
| | 24.2 | 73.21 ± 10.13 | 75.42 ± 13.37 | 73.69 ± 18.32*** | 67.71 ± 17.69*** |

Results are presented as mean ± SD of the wound width, n = 8-12 at various time points ($T_0$, 4 hours ($T_4$), 24 hours ($T_{24}$), and 48 hours ($T_{48}$)). Asterisks represent statistically significant changes versus control (* $p < 0.05$; ** $p < 0.01$; *** $p < 0.001$).

**Table S5.** The effects of metformin and its sulfonamide derivatives on MDA-MB-231 cell migration.

| Compound | Conc. [μmol/L] | $T_0$ [μm] | $T_4$ [μm] | $T_{24}$ [μm] | $T_{48}$ [μm] |
|---|---|---|---|---|---|
| **Control** | - | 84.73 ± 11.95 | 75.38 ± 11.82 | 55.42 ± 10.66 | 35.01 ± 9.28 |
| **Metformin** | 500 | 83.26 ± 3.90 | 78.51 ± 5.50 | 72.44 ± 5.35*** | 62.87 ± 4.62*** |
| | 1000 | 80.97 ± 4.53 | 76.91 ± 2.95 | 71.10 ± 2.43*** | 64.74 ± 5.04*** |
| **Comp. 4** | 327 | 85.89 ± 5.09 | 80.13 ± 4.79 | 49.57 ± 8.52 | 36.09 ± 9.91 |
| | 654 | 80.50 ± 7.82 | 74.86 ± 9.03 | 50.14 ± 11.35 | 41.25 ± 14.28 |
| **Comp. 8** | 226 | 87.88 ± 9.50 | 82.53 ± 13.39 | 59.55 ± 7.19 | 52.73 ± 11.76*** |
| | 452 | 80.20 ± 15.48 | 77.14 ± 11.12 | 65.09 ± 9.72** | 50.01 ± 13.74*** |
| **Comp. 10** | 248 | 91.03 ± 11.53 | 89.98 ± 9.28** | 69.47 ± 7.39*** | 58.85 ± 13.74*** |
| | 496 | 83.63 ± 12.41 | 87.55 ± 13.48** | 65.35 ± 10.54** | 56.99 ± 15.74*** |

Results are presented as mean ± SD of the wound width, n = 8-12 at various time points ($T_0$, 4 hours ($T_4$), 24 hours ($T_{24}$), and 48 hours ($T_{48}$)). Asterisk represent statistically significant changes versus control (* $p < 0.05$; ** $p < 0.01$; *** $p < 0.001$).

**Figure S4.** Inhibition of cell migration in the presence of selected biguanides. MCF-7 (A) and MDA-MB-231 (B) cells migration was evaluated using wound healing assay. Representative cell images are shown for control samples and biguanides at the concentrations corresponding to 1/2x$IC_{50}$ value. Cells were photographed using an inverted microscope at the indicated times (t = 0, 4, 24 and 48 h); 100 × magnification.

A)

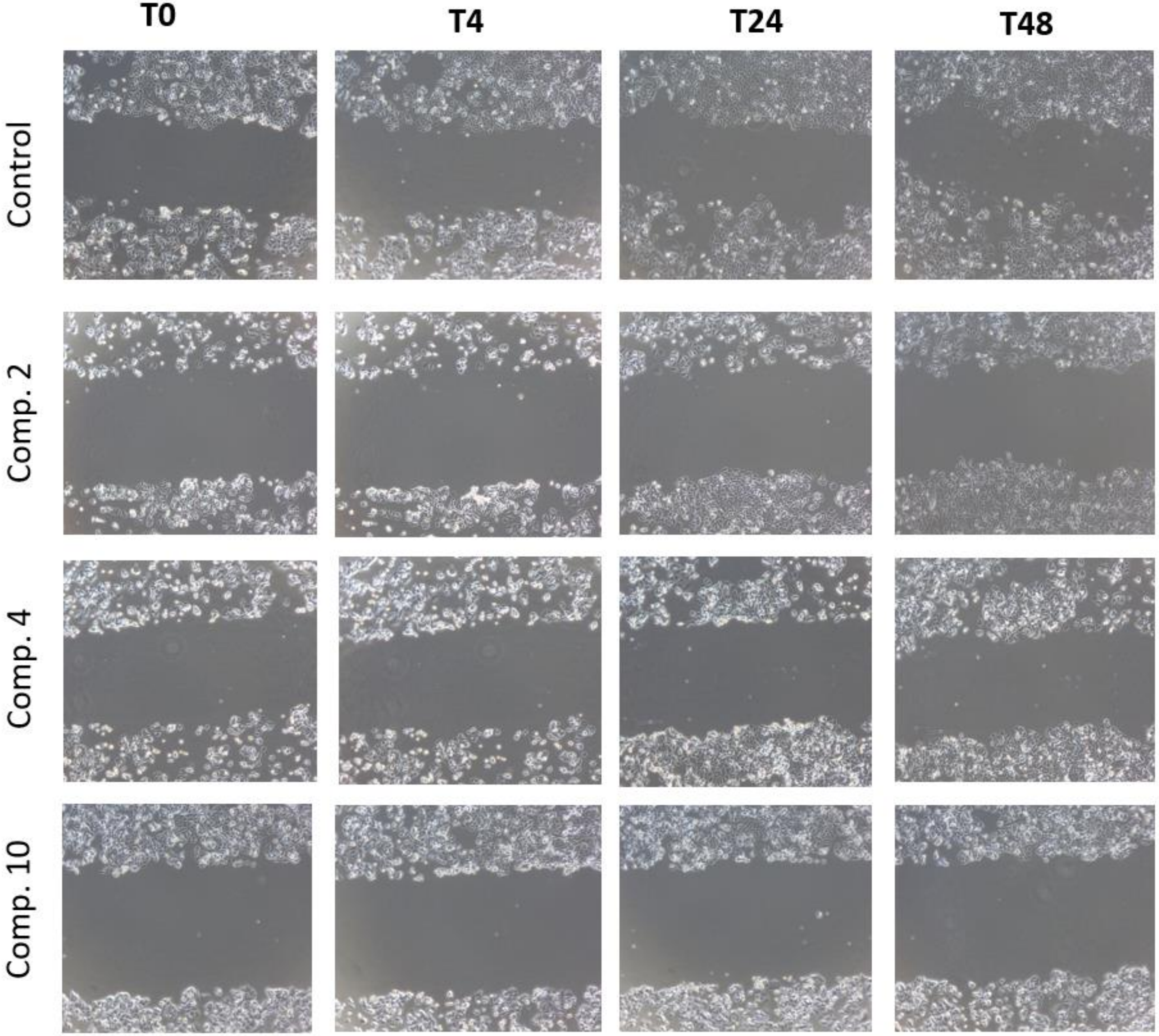

B)

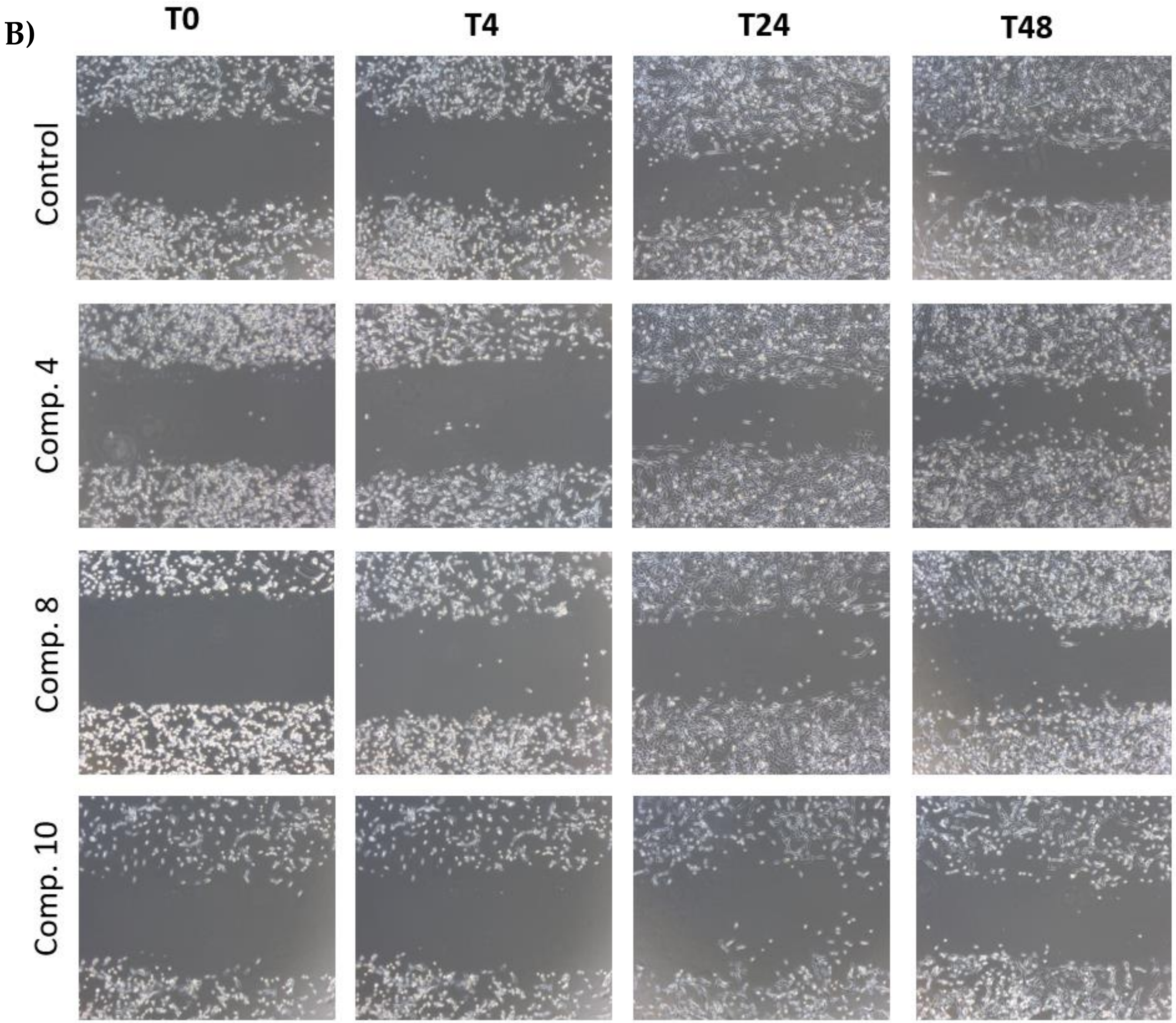

Supplement: Supplementary file 1 [file ijms-22-05642-s001.zip › ijms-1222880-supplementary.pdf]
